# The nature of the post-translational modifications of the autoantigen LL37 influences the autoreactive T-helper cell phenotype in psoriasis

**DOI:** 10.3389/fimmu.2025.1546422

**Published:** 2025-04-09

**Authors:** Roberto Lande, Anna Mennella, Raffaella Palazzo, Rebecca Favaro, Paola Facheris, Flavia Mancini, Giuseppe Ocone, Elisabetta Botti, Mario Falchi, Immacolata Pietraforte, Curdin Conrad, Luca Bianchi, Antonio Costanzo, Loredana Frasca

**Affiliations:** ^1^ Istituto Superiore di Sanità, National Center for Global Health, Roma, Italy; ^2^ Department of Biomedical Sciences, Humanitas University, Pieve Emanuele, Milan, Italy; ^3^ Dermatology Unit, Istituto di Ricovero e Cura a Carattere Scientifico (IRCCS) Humanitas Research Hospital, Rozzano, Milan, Italy; ^4^ Dermatology Unit, Department of Systems Medicine, University of Rome Tor Vergata, Rome, Italy; ^5^ Istituto superiore di Sanità, National AIDS Center, Rome, Italy; ^6^ Istituto Superiore di Sanità, Department of Oncology and Molecular Medicine, Rome, Italy; ^7^ Department of Dermatology, University Hospital of Lausanne (CHUV), Lausanne, Switzerland

**Keywords:** psoriasis, autoreactive responses, carbamylation, citrullination, autoantigens, LL37, T helper cell polarization

## Abstract

Psoriasis is a chronic skin disease evolving to psoriatic arthritis (PsA) in 30% of cases. LL37 is a psoriasis T-cell autoantigen and, in complex with self-DNA/RNA, a trigger of type I interferon (IFN-I) and pro-inflammatory factors in dendritic cells. LL37 can undergo irreversible post-translational modifications (PTMs), namely, citrullination and carbamylation, which are linked to a neutrophil-dominated inflammation. Notably, in PsA, carbamylated and citrullinated LL37 (carb-LL37 and cit-LL37) become antibody targets. Here, we analyze the presence of, and the T-cell and antibody reactivity to, cit-LL37 and carb-LL37, to address the occurrence and significance of these PTMs in psoriasis. The presence of modified LL37 in skin biopsies was assessed by laser scanner confocal microscopy (LSCM); T-cell responses to modified LL37 were assessed by Ki67 assay and intracellular cytokine staining using flow cytometry; serum autoantibodies to the same antigens were tested by enzyme-linked immunosorbent assay (ELISA). The results show that native and modified LL37 (both carb-LL37 and cit-LL37) are detectable in psoriatic skin, but not in healthy donors’ (HD) skin, where they colocalize with neutrophil infiltrates and neutrophil extracellular trap formation (NETosis). Psoriatic T cells and antibodies recognize native LL37, cit-LL37, and carb-LL37, but only CD4-T-cell responses to native LL37 and carb-LL37 correlate with psoriasis area severity index (PASI), whereas CD8-T-cell responses to the same peptides correlate with PASI in the HLA-Cw6*02-positive subgroup. CD4-T cells specific for modified LL37 express heterogeneous T-helper (Th) phenotypes: native/carb-LL37-specific T cells mainly manifest a Th1/Th17-like phenotype, whereas cit-LL37-specific T cells resemble Th-follicular (Thf)-like cells. *In vitro* T-cell polarization experiments suggest that distinct pro-inflammatory effects of LL37 and modified LL37, in complex with self-nucleic acids, may concur to these phenomena. This is the first evidence in psoriasis that PTMs of an autoantigen with innate immune cell stimulatory ability dictate autoreactive Th-cell polarization. These data, obtained using LL37 as a model autoantigen, indicate that citrullination and carbamylation pathways may play a role in the psoriasis course, generating epitopes to which immunological tolerance does not exist and potentially concur to PsA development.

## 1 Introduction

Psoriasis is a chronic inflammatory and autoimmune skin disease affecting up to 4% of individuals worldwide (1–5). Inflammatory cytokines produced by pathogenic T-helper 1 (Th1)/Th17 cells, interleukin-17 (IL-17), tumor necrosis factor-α (TNF-α), interferon-γ (IFN-γ), IL-22, and IL-21 sustain chronic inflammation and/or hyper-proliferation of keratinocytes (1–14). T cells and T-cell-derived cytokines have long been known to contribute significantly to psoriasis pathogenesis although the autoantigens they recognized were only in part understood (15, 16). We demonstrated that the antimicrobial peptide (AMP) cathelicidin LL37 acts as a psoriasis autoantigen (17). Being efficiently presented by multiple HLA-DR alleles and by the psoriasis-associated MHC-I molecule HLA-Cw6*02 (1–3, 17), LL37 was found to be recognized by T cells in up to 40% of the patients with psoriasis. Notably, 30% of patients with psoriasis develop a severe form of arthritis [psoriatic arthritis (PsA)], and we have recently demonstrated that T cells of patients with PsA also recognize LL37 as an autoantigen (18). Moreover, anti-LL37 autoantibodies and antibodies recognizing cit-LL37 and carb-LL37 were present in inflamed PsA synovia and in circulation (18). These observations suggest that pathways leading to LL37 carbamylation and citrullination are activated in PsA and can play a pathogenic role. However, the presence of cit-LL37 and carb-LL37 in psoriasis skin and its relationship with T-cell activation and with the occasional presence of anti-LL37-specific antibodies are unclear. As hypothesized in rheumatoid arthritis (RA), PsA, and systemic lupus erythematosus (SLE), the neutrophilic inflammation could be viewed as a bridge between innate and adaptive immunity, via promotion of those post-translational modifications (PTMs) that favor the break of tolerance to self-antigens, and this is the case for carbamylation and citrullination (18–23). We demonstrated that epitopes of LL37 that are citrullinated or carbamylated can be presented to, and are immunogenic for, SLE CD4 T cells; SLE is a chronic disease with a recognized neutrophilic signature (20–22). Knowing that neutrophils can also infiltrate psoriatic skin (24, 25), and carbamylation and citrullination may occur locally in the presence of neutrophils either degranulating or undergoing neutrophil extracellular traps extrusion (NETosis), the purpose of this study was to address whether LL37, modified by citrullination and carbamylation, might concur to the pathogenesis of psoriasis, by affecting the characteristics of autoreactive T cells. This might revel the close interplay between neutrophils and autoreactivity in psoriasis. Indeed, we have previously demonstrated that LL37 modified by citrullination and carbamylation differs in its capacity to trigger IFN-I in plasmacytoid dendritic cells (pDCs) (22). Thus, here we have analyzed the occurrence of citrullination and carbamylation in psoriasis lesional skin, and the effect of these modifications on adaptive immunity activation, using LL37 as model autoantigen (17). LL37-specific T cells, of both CD4 and CD8 phenotype, appear to coexist with T cells that recognize the modified LL37 versions. This finding indicates a multi-specific T-cell response in psoriasis, and perhaps cross-reactivity of cit-LL37- and especially carb-LL37-specific T cells with the native antigen. Moreover, the nature of the autoantigen modification, which is likely to have an impact on the “adjuvant activity” of native LL37, appears to influence the fate of the psoriasis CD4 T cells with respect to T-helper cell polarization outcomes and, likely, effector functions.

## 2 Materials and methods

### 2.1 Study design

The blood of patients with psoriasis and healthy donors (HD) was obtained from Humanitas Hospital, Milan, Italy (**Supplementary Table S1**). The skin biopsies of both patients with psoriasis and HD were from University of Tor Vergata, Rome. As psoriasis score, we used psoriasis area severity index (PASI) as previously published (17). Patients with psoriasis did not take medications in the last 3 months before first blood sampling. All samples were obtained after approval by the Ethic Committees of Humanitas (ICH Ethic Committee, Rozzano, MI) and the University of Tor Vergata, Rome. All blood and tissue donors gave informed consent. Patients were genotyped for the presence of the MHC-I allele HLA-Cw6*02: 20 ng of DNA extracted from 0.2 mL of blood (Qiagen 51104) was tested for the presence on a 3% agarose gel of the PCR-specific 304-bp product amplified with primers [HLA-Cw06F 5′-TACTACAACCAGAGCGAGGA-3′; HLA-Cw06R 5′-GGTCGCAGCCATACATCCA-3′ Bio-Rad Master Mix for PCR (1665009EDU)].

### 2.2 Antigens

LL37: (LLGDFFRKSKEKIGKEFKRIVQRIKDFLRNLVPRTES) was purchased from Proteogenix (Schiltigheim, Strasbourg, France). Reverse (REV) LL37 (SETRPVLNRLFDKIRQVIRKEFEKGIKEKSKRFFDGLL); citrullinated LL37 (cit-LL37) {LLGDFFR(cit)KSKEKIGKEFKR(cit)IVQR(cit)IKDFLR(cit)NLVPR(cit)TES}; citrullinated reverse (cit-REV) {SETR(cit)PVLNR(cit)LFDKIR(cit)QVIR(cit)KEFEKGIKEKSKR(cit)FFDGLL}; carbamylated LL37 (carb-LL37)(L*LGDFFRK*SK*EK*IGKEFK*RIVQRIK*DFLRNLVPRTES), in which the asterisks indicate substitution with homocitrullines; and carb-REV: (SETRPVLNRLFDK*IRQVIRKEFEK*GIK*EK*SK*RFFDGLL*), in which the asterisks indicate substitution with homocitrullines, were all synthesized by Biomatik (Wilmington, DE, USA).

### 2.3 Antibodies

Antibodies to CD4, CD3 (UCHT1), CD1, CD14, and CD1a were used conjugated with various fluorochromes [FITC, phycoerythrin (PE) PerC, peridinin-chlorophyll-protein (PerCp), allophycocyanin (APC), PE-Cy7, and Pacific Orange], and were from BD Biosciences or eBiosciences (San Diego, CA). For further LL37-specific T-cell characterization, we used the following: APC-Cy7-CXCR5 (CD185, FAB190), APC or PE or Pacific blueT (PB)-CD38 (clone: HB-7), PD1 conjugated with PE/Dazzle (CD279), and Bcl-6 (APC conjugated), purchased from BD Biosciences, eBiosciences, Novus Biologicals (Littleton, CO), and R&D (Minneapolis, MN). APC or AlexaFluor 700-conjugated anti-Ki67 antibody (BD Pharmingen, SolA15) was used in combination with anti-IL-17 (IL-17A) PB, anti-IL-21 AlexaFluor 647, and IFN-γ. Appropriate isotype-matched controls were from the same companies. The following were the antibody clones used: anti-CD3, clone UCHT1; anti-CD4, clone OKT4; anti-CXCR5, clone J252D4; anti-CD38, clone HB-7; anti-PD1, clone EH12.2H7; anti-BCL6, clone BCL-UP; anti-IL17, clone BL168; anti-IL21, clone 3A3-N2: anti-IFN-γ, clone B27; anti-Ki67, clone solA15; anti-human T-bet, clone 4B10; and anti-MPO, clone EPR20257. The rabbit monoclonal antibody specific for carb-LL37 (clone RRB640) and the mouse antibody specific for cit-LL37 (Mab142), not cross-reacting to native/carb-LL37 and cit-LL37, were obtained in the Antibody Facility of UNIGE, Geneva, CH (22). The rat anti-myeloperoxidase (MPO) monoclonal antibody (Rat IgG2a) was from Abcam (UK, ab300651). Secondary anti-mouse antibodies conjugated with AlexaFluor 647, AlexaFluor 488, or AlexaFluor 555 were from Abcam (UK). Isotype controls were from Hycult (Sweden).

### 2.4 Measurement of antibody reactivity to native LL37, cit-LL37, and carb-LL37 in sera of patients with psoriasis and HD

Anti-native/cit-LL37/carb-LL37 antibody reactivity was measured by enzyme-linked immunosorbent assay (ELISA) as previously described (21, 22). Briefly, 96-well flat-bottom plates (non-binding surface polystyrene, Corning, USA) are coated with 2 μg/mL of native LL37/cit-LL37/carb-LL37 or control proteins (cit-REV and carb-REV) in carbonate buffer (0.1 M NaHCHO_3_, pH 9) for 2 h (or overnight) and washed four times with PBS 0.1% Tween-20. This washing buffer was used for washing at all steps. The blocking buffer containing 2% bovine serum albumin (BSA, Sigma) in PBS was used for at least 1 h (or overnight) to saturate unspecific binding sites. After washing, sera were diluted at various concentrations (usually 1:100) in PBS 2% BSA followed by 1-h incubation with a horseradish peroxidase (HRP)-conjugated goat anti-human IgG (Sigma-Aldrich), diluted 1:10,000 in PBS. The color was developed for 5 min with 3,3′,5,5′-tetramethylbenzidine (TMB) substrate (Sigma-Aldrich). The reaction was stopped by adding 50 μL of 2 N H_2_SO_4_, and absorbance was determined at 450 nm with a reference wavelength of 540 nm. The positivity for antibodies to LL37, cit-LL37, and carb-LL37 was considered present when the OD measured in response to these antigens was above a cutoff obtained by the mean of OD measured with HD plasma/sera plus two standard deviations. In addition, if reactivity to LL37, cit-LL37, and carb-LL37 was found in the presence of significant reactivity to REV, cit-REV, or carb-REV, these patients were not considered to be positive for these the anti-LL37 and modified LL37 antibodies (21, 22).

### 2.5 Measurement of proliferation, marker expression, and cytokine production in primary T cells

PBMCs were purified from EDTA-treated blood, on Ficoll-Hypaque (Pharmacia Fine Chemicals, Uppsala, Sweden) and were incubated (10^5^ cells/well) in 96-well flat-bottom microplates (BD) in T-cell medium [RPMI 1640, 10% heat-inactivated human serum (HS), Gibco, 2 mM L-glutamine, 10 U/mL penicillin, and 100 μg/mL streptomycin], with/without peptides. We performed assays on PBMCs frozen in 90% FCS–10% DMSO, whose viability we assessed by Trypan blue exclusion, with an inverted microscope. Recovery of live cells was between 65% and 85% of the frozen number. Proliferation was assessed by Ki67 staining (intracellular staining performed after the surface staining), using APC-labeled anti-Ki67 antibody (BD Pharmingen), after 5 days of culture and activation with phorbol myristate acetate (PMA) (Sigma)/ionomycin (Calbiochem) (PMA+Iono at 50 ng/mL and 1 μg/mL, respectively), for 4 h. Secretion was blocked with Brefeldin A (Selleckchem, USA, at 10 μg/mL added after the first 30 min with PMA+Iono). Aqua dye (LIVE/DEAD™ Fixable Aqua Dead Cell Stain Kit, Thermo Fisher) was added to exclude dead cells into the assay. Internal control for T-cell viability was PHA treatment (2 μg/mL). Phenotype analysis of LL37-responder T cells (Ki67-positive cells, at 5 days of culture) included staining for CD3, CD4, and CXCR5, and for cytokines (with anti-IFN-γ, -IL-17, and -IL-21 antibodies, of different colors, as above). Cells were permeabilized and fixed by using the Intracellular fixation/permeabilizazion buffer set (eBiosciences). T cells were gated on CD3/CD4 or CD3/non-CD4 (CD8) markers and cytokine-staining analyzed on Ki67^+^ cells. Cells were analyzed by flow cytometry using a Gallios, BeckmanCoulter (Brea, CA, USA).

Stimulation index (SI) for proliferation was calculated by dividing the percentage of Ki67-staining (percent of positive T cells) in the presence of each peptide antigen (LL37, carb-LL37, and cit-LL37) by the percent obtained with the control antigens (LL37 reverse, REV; cit-LL37 reverse, cit-REV; carb-LL37 reverse, and carb-REV). SI for proliferation was considered positive when the values of the percentage of Ki67-positive cells were three times higher than the values obtained with the control peptides (SI > 3). This cutoff was obtained by calculating the mean ± 2 SD (of the SIs calculated as above) of the proliferation of HD. Basal % of proliferation was set using, as reference, an isotype-matched antibody staining (AlexaFluor 700 isotype control) and by never exceeding 5% of the cell proliferation value (background proliferation). Concomitant phenotype analysis of LL37-responder T cells included staining for CD4, CXCR5, PD1, and Bcl-6 (BCL-UP); all were measured by intracellular staining and flow cytometry, after 48-h culture with LL37 or control antigen, and were analyzed on CD38^high^ (activated) CD4 T cells. SI for expression of CXCR5, Bcl-6, and cytokines was calculated as the percentage of the Ki67^+^CD3^+^CD4^+^ cells that expressed the specific marker or cytokine, in the presence of the peptide antigen, with respect to the same values obtained after stimulation with control peptides.

### 2.6 Staining of skin biopsies and laser scanner confocal microscopy for analysis of the skin

We performed staining on 6-mm paraffin or frozen sections of psoriasis skin specimens (or from control HD), obtained from University of Tor Vergata, Rome. Biopsies in paraffin were stained after de-paraffination in xylene (5 min, two times), followed by passages in absolute ethanol (3 min), 95% ethanol in water (3 min), 80% ethanol in water (3 min), 70% ethanol in water (3 min), and antigen retrieval (5 min at 95°C in 10 mM sodium citrate, pH 6.0). Slides were saturated with a solution containing 4% BSA for 1 h. After washing three times for 3 min under agitation with PBS 0.1% Tween 20, the slides were stained for cit-LL37 and carb-LL37 using the mouse antibody Mab142 (specific for cit-LL37), and the rabbit monoclonal antibody specific for carb-LL37 (clone RRB640), at 1:40 dilution, respectively. MPO was detected using the anti-MPO antibody, Rat IgG2a-300651 (Abcam). Slides were incubated in a humidified chamber, for 1 h. After washing as above, we incubated the slides with a donkey anti-mouse antibody (AlexaFluor 647), a donkey anti-rabbit antibody conjugated with AlexaFluor 488, and a goat anti-rat antibody conjugated with AlexaFluor 555 (all from Sigma), again in a humidified chamber, for 1 h. Slides were washed again, as above, and mounted in Prolong Gold anti-fade media containing a DNA dye (DAPI) (Molecular Probes), before analysis. Images were taken on a confocal microscope Zeiss LSM 900 (Carl Zeiss GmbH, Jena, Germany) in Airyscan mode. Excitation light was obtained by diode lasers: 405, 488, and 548 nm. Optical thickness was 0.20 µm with 63× objective (Zeiss, AN 1.20). A semi-automatic evaluation of colocalization of staining between two markers of interest was performed by measuring the percentage of relative area occupied, in each biopsy, by the overlay of the two colors considered (visualized either in magenta or in yellow in some processed images). These values were obtained selecting one stack in each scanned biopsy. Images have been treated and analyzed by the Zen Blue (3.2) software (Carl Zeiss GmbH, Jena Germany).

### 2.7 Extraction of human RNA

Human RNA was extracted from PBMCs, as previously reported (26). The resulting size distribution was controlled by 2% agarose gel electrophoresis.

### 2.8 Production of monocyte-derived DC and stimulation/polarization of CD4 T cells

Monocytes were purified as previously described (26), using the CD14 MicroBeads human kit Miltenyi Biotec (Germany) and cultured at 10^6^/mL in complete medium in the presence of 500 U/mL of hrIL-4 (Cell Guidance Systems, CellGS, St. Louis, USA) and 50 ng/mL rGM-CSF (Peprotech, Thermo Fisher Scientific, USA). After 6 days, monocyte-derived dendritic cells (MDDCs) were harvested and used as APC in Ki67 assays. Cells were checked for the expression of CD1a and CD14 expression and were at least 95% pure. Differentiated MDDCs were CD1a-positive and CD4-low or -negative as previously described (26, 27). MDDC-induced allogeneic T-cell proliferation (PBMC/MDDC ratio 10:1) was assessed after 7 days of culture in the presence or absence of different concentrations of IFN-α (IFN-α4 and IFN-α2 proteins, Bio-Techne, Minneapolis, MN, USA); the two IFN-α types were added together to the cultures to reach the final concentration of 1,000 UI/mL or 300 UI/mL. In additional experiments, immature MDDCs were treated overnight with LL37 (1 μM), cit-LL37 (1 μM), and carb-LL37 (1 μM), alone or in complex with human RNA (15 μg/mL), plus/minus IFN-I (1,000 U/mL, as above). CD4 T cells were isolated using the human pan T-cell isolation human kit, followed by the use of the human CD4^+^ T-cell isolation kit (both by Miltenyi, Germany). Proliferation and phenotype of responder CD4 T cells cultured with the MDDC were assessed by Ki67 intracellular staining and by surface staining with anti-CXCR5 and anti-PD1 antibodies, respectively, after a 6-day culture. Part of the supernatants from overnight cultured MDDCs, preceding the CD4 T-cell-MDDC culture setup, were harvested and tested for the presence of TNF-α, IL-23, and IL-12, by ELISA (MabTech, France). IL-17 and IFN-γ concentrations in the supernatants of the CD4 T cells cultured with the MDDC were also measured by ELISA (MabTech, France), at day 6 of culture. All ELISAs were performed according to the manufacturer’s instructions.

### 2.9 In-house ELISA for the determination of blood myeloperoxidase–DNA complexes

MPO–DNA complexes were identified using a capture ELISA. The capturing antibody, 2 μg/mL, was a rat anti-human MPO antibody (anti-MPO antibody, 300651, Abcam). The anti-MPO antibody was coated to 96-well plates (100 μL), overnight at room temperature. After blocking in PBS 2% BSA (200 μL), plasma (100 μL diluted to 1:50 in 1% BSA in PBS) was added and incubated for 2 h at room temperature. After incubation, wells were washed four times with 200 μL of 0.05% Tween 20 in PBS, and the mouse anti-human dsDNA (Abcam, 1:300) was added for 1 h at room temperature. After washing, wells were incubated for 1 h with an HRP-conjugated rabbit anti-mouse IgG (SouthernBiotech) (dilution 1:600 in PBS). After washing, the chromogenic substrate TMB was added and incubated in the dark. The absorbance was measured at 450 nm after stopping the reaction as above. Plasma samples were considered positive when the OD was above an established cutoff, which was calculated as the mean plus two times the standard deviation of the OD values obtained with HD plasma samples.

### 2.10 Statistical analyses

Differences between mean values were assessed by Wilcoxon’s matched-pair signed rank test, or paired *t*-test, to compare responses to antigens and control antigens in the same donor. We used Mann–Whitney or unpaired *t*-test for comparison of T-cell and antibody responses between groups of patients and HD. Statistical significance was set at *p* < 0.05. Correlation analyses were performed by Spearman’s (for low sample size) and Pearson’s rank-correlation test (for higher sample size). Data were analyzed by GraphPad Prism 7.0 or SPSS software.

## 3 Results

### 3.1 Carb-LL37 and cit-LL37 are detected in psoriasis skin in the presence of neutrophils

**Figure 1:**
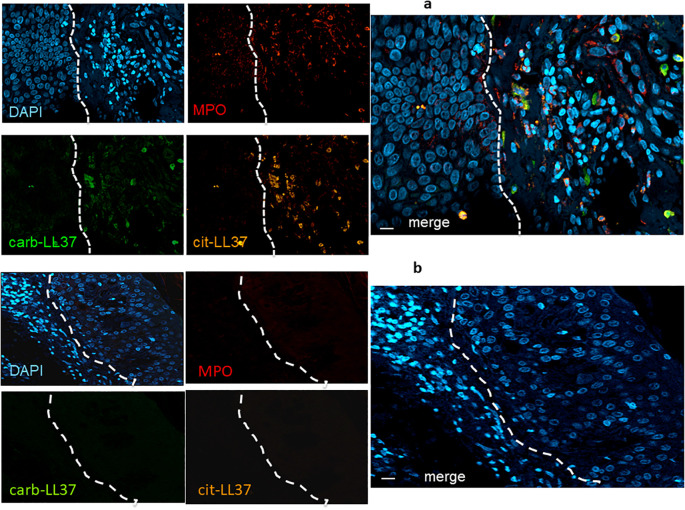
Cit-LL37 and carb-LL37, together with MPO, are expressed in psoriasis dermis. Skin biopsies of patients with psoriasis **(a)** and of HD **(b)** were stained with the mouse anti-cit-LL37 (Mab142, orange), the rabbit anti-carb-LL37 (RRB640, green), or a rat anti-human MPO (red). Cell nuclei were stained with DAPI. Biopsies were analyzed by LSCM using a specific microscope (see Materials and Methods). Results are representative of six psoriasis and four HD biopsies stained and analyzed in the same way. The dotted lines, in white, indicate the separation of the epidermis [left part of each picture, in **(a)** and right part of each picture in **(b)**] from the dermis. MPO, cit-LL37, and carb-LL37 are only expressed in the dermis part of each picture. Objective, 40×; bar, 10 μm.

**Figure 2:**
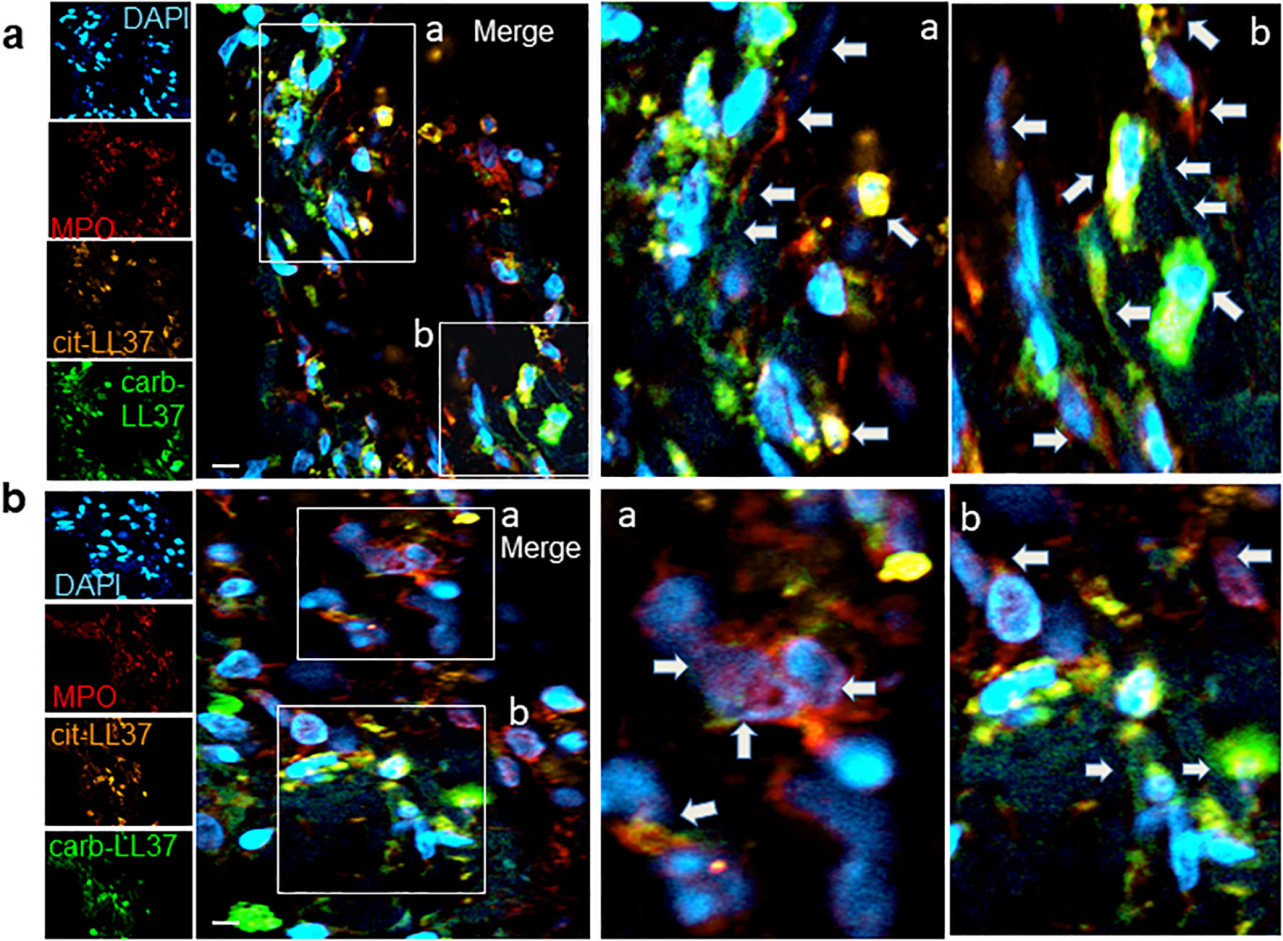
NETosis occurs in psoriasis dermis and NET colocalizes with expression of cit-LL37, carb-LL37, and MPO. Skin biopsies of patients with psoriasis **(a, b)** were stained with the mouse anti-cit-LL37 (Mab142, orange), rabbit anti-carb-LL37 (RRB640, green), or with rat anti-human MPO (red). Cell nuclei were stained with DAPI. The pictures represent the dermis part of the skin, where MPO, cit-LL37, and carb-LL37 are expressed (as in **Figure 1**). Results obtained are representative of four psoriasis skin biopsies analyzed as in **Figure 1**. Pictures in **(a, b)** are from two different patients with psoriasis. The arrows indicate the expression of MPO or modified LL37 in the same areas or colocalizing with each other, for example, as in insets a and b of **(b)** (see also colocalization in **Supplementary Figure S2**); in some cases [as in insets a and b of **(a)**], DNA filaments decorated by MPO and/or carb-LL37 are also evidenced by the arrows. Objective, 63×; bar, 10 μm..

**Figure 3:**
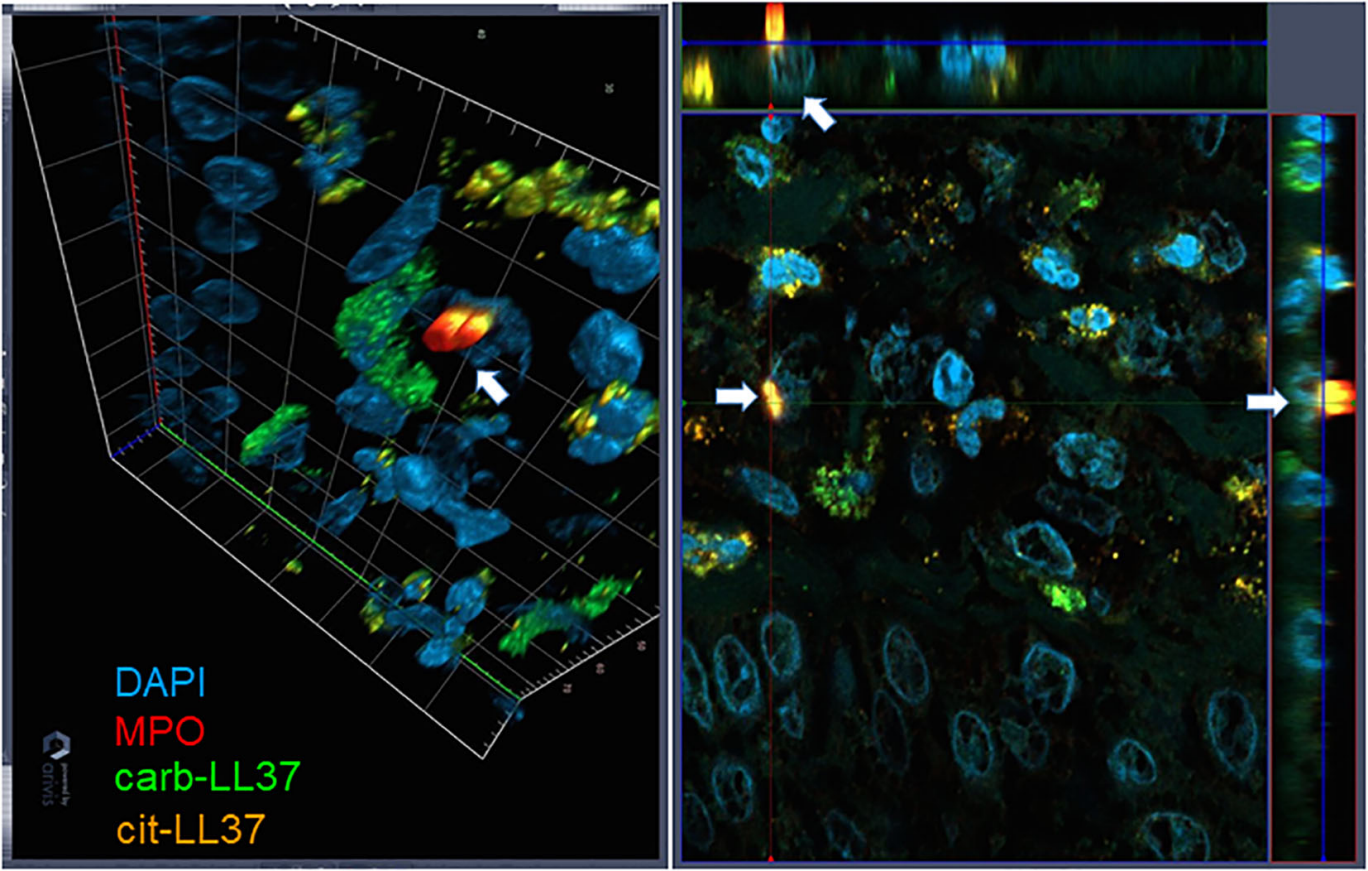
Three-dimensional representation of NET. Skin biopsies were stained as in **Figures 1** and **2**. The picture on the left represents a tridimensional psoriasis skin (dermis), in which one cell, indicated with the white arrow, is extruding NET. On the right, the same image is projected into the three-space direction, and the arrows represent the cell extruding NET in these three dimensions. Results are representative of two psoriasis biopsies analyzed as in **Figures 1** and **2**. Objective, 63×.

Knowing that native LL37 is highly expressed in psoriatic plaques (17, 28), we wondered whether cit-LL37 and carb-LL37 peptides were expressed in psoriasis skin. We took advantage of newly developed monoclonal antibodies, specific for cit-LL37 and carb-LL37 (22, 29), which do not cross-react to native LL37, to address the presence of cit-LL37 and carb-LL37 in psoriasis plaques by laser scanner confocal microscopy (LSCM). Moreover, we stained for MPO expression to reveal neutrophil infiltrates. MPO is one of the markers of neutrophils, although alternative markers could be preferred by some authors to identify neutrophil extracellular trap (NET) structures (30–32). Here, we preferred to assess MPO presence because of its role in carbamylation. MPO presence can be due to NETosis, but can also be NETosis-independent (33, 34). Cit-LL37 and carb-LL37 were detectable in the lesional psoriasis skin in the presence of MPO expression (**Figure 1a**), suggesting that proteins released by neutrophils could be responsible for both PTMs. The expression of both modified LL37 forms was mostly confined to the dermis. Where neutrophils were scarce or absent, cit-LL37 and carb-LL37 were almost negative or confined to the zone with MPO expression (**Supplementary Figure S1**). HD biopsies were negative for LL37, cit-L37, carb-LL37, and MPO staining (**Figure 1b**). As shown in **Figure 2**, NET structures were detectable in the psoriasis dermis, where structures similar to DNA filaments decorated by MPO or modified LL37 were observed. Both cit-LL37 and carb-LL37 mostly colocalized with MPO staining (**Figure 2**, **Supplementary Figure S2**). This is also evident, at the cell level, in **Figure 3**, which depicts the three-dimensional distribution of NET and the colocalization of cit-LL37, carb-LL37, and MPO in the single cell that is extruding material. **Supplementary Figure S2** reports a calculation, by confocal microscopy, of the relative percent area of biopsies from patients with psoriasis and HD occupied by MPO colocalizing with either carb-LL37 or cit-LL37 (see Materials and Methods). These results suggest that cit-LL37 and carb-LL37 colocalized with, or were expressed in proximity of, MPO staining. These results indicate that cit-LL37 and carb-LL37 were mainly detectable in those skin biopsies where neutrophils infiltrated the dermis and in the presence of NET. Given this, we analyzed whether circulating MPO–DNA immune complexes (indicating NET formation *in vivo*) were present in our cohort of patients with psoriasis (see Materials and Methods). A previous study indicated that patients with psoriasis might harbor MPO–DNA complexes in circulation, which suggested that NETosis could occur in patients (35). We checked the expression of MPO–DNA complexes in the blood of our patients with psoriasis as compared to HD and we found a higher expression in patients (*p* < 0.0001, **Supplementary Figure S3a**). Moreover, the presence of MPO–DNA complexes (when only the patients that were positive were considered, *N* = 63, 65%) correlated positively with the PASI (Spearman’s *r* = 0.25, *p* = 0.026, *N* = 63) (**Supplementary Figure S3b**). Overall, these data suggest that carb-LL37 and cit-LL37 may be generated in inflamed psoriasis skin when a neutrophil infiltrate is present and NET is produced.

### 3.2 T-cell proliferation to modified LL37 occurs in psoriasis

**Figure 4:**
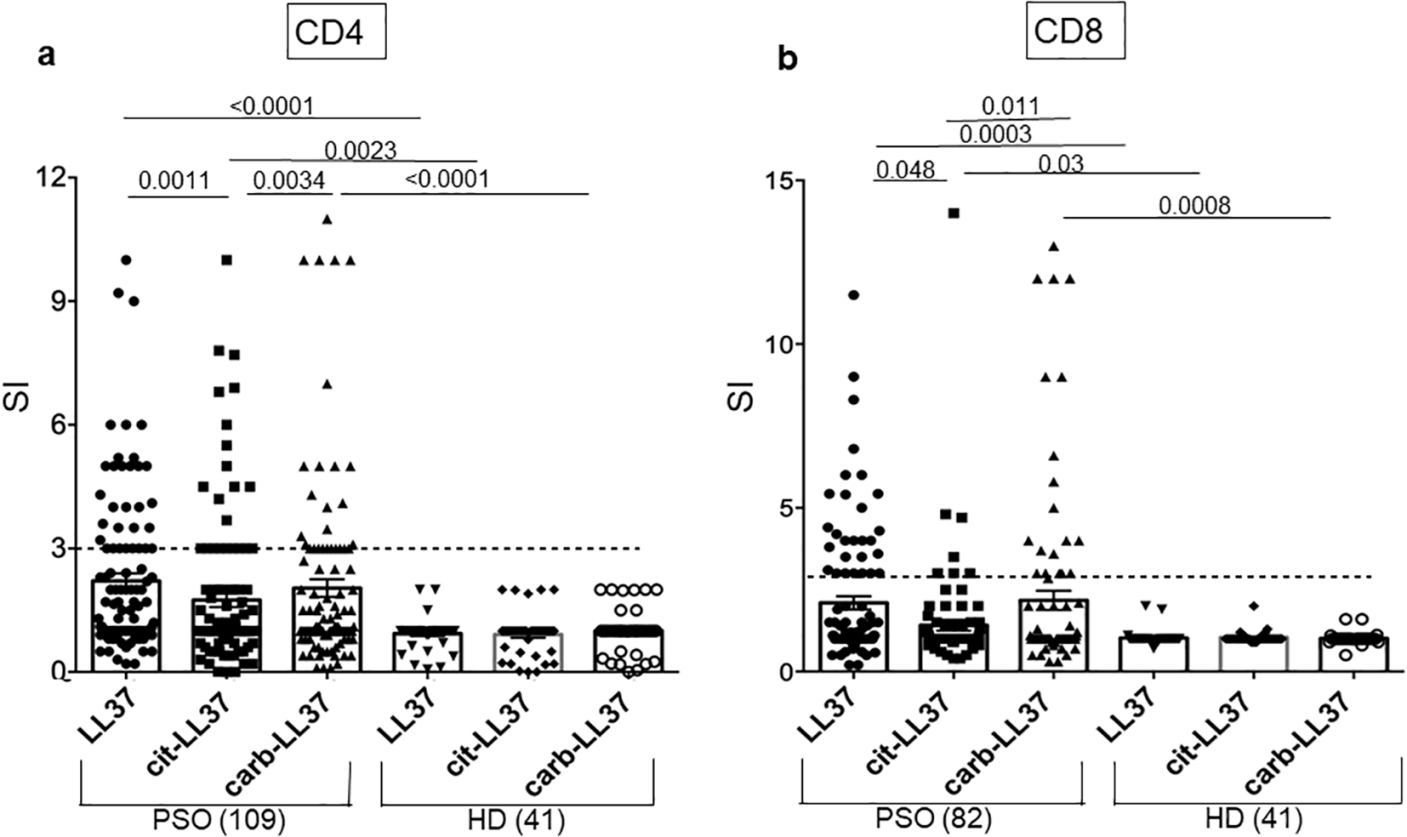
T-cell proliferation to native LL37, cit-LL37, and carb-LL37 occurs in patients with psoriasis. Cumulative results of Ki67 assays for assessing the proliferation of CD4 T cell **(a)** or CD8 T cell **(b)** to LL37 and modified LL37. All results are expressed as stimulation indexes (SIs), as indicated in Materials and Methods. Proliferation was assessed by flow cytometry as percentage of Ki67^+^ cells in the gate of CD3^+^CD4^+^ cells (CD4-T cells) or in the gate of CD3^+^CD4^−^ cells (CD8 T cells) in response to each peptide (see the gating strategy in **Supplementary Figure S4**). In the graphs, horizontal small bars represent the means, vertical bars are the standard errors of the mean, and *p*-values are from the Mann–Whitney test. Number of patients and HD tested are indicated in the graphs. The horizontal dotted lines represent the cutoff that distinguishes positive T-cell responses from negative responses.

Next, we assessed T-cell reactivity to cit-LL37 and carb-LL37, as compared to the responses to the native LL37. We used a cell proliferation assay based on Ki67 staining (see **Supplementary Figure S4** for gating strategy for both CD4 and CD8 T-cell proliferation). **Figure 4** reports T-cell proliferation as cumulative data. Proliferation is expressed as SIs (see Materials and Methods). CD4 T cells of 54 out of 109 patients with psoriasis (50%) responded to any of the LL37 form; 36 out of 109 (33%) responded to native LL37, 27 out of 109 (25%) responded to cit-LL37, and 34 out of 109 (31%) responded to carb-LL37 (**Figure 4a**). HD (*N* = 41) did not significantly respond to any of these peptides. The median of SI of T-cell responses to carb-LL37 was significantly higher than that of responses to cit-LL37 (*p* = 0.0034) (**Figure 4a**). Results of CD8 T-cell responses were available in 82 patients with psoriasis of the studied cohort (**Figure 4b**): we found that 29 out 82 (35%) patients responded to native LL37, 7 out of 82 (9%) responded to cit-LL37, and 19 out 82 (23%) responded to carb-LL37 (**Figure 4b**). In the cohort, 24 patients with psoriasis plus PsA were also present, and they did respond to LL37 and modified LL37 [CD4-T-cell responses: 9 out of 24 responded to native LL37 and cit-LL37 (38%) and 8 out of 24 responded to carb-LL37 (33%); CD8-T-cell responses: 6 out of 24 responded to native LL37 and carb-LL37 (25%) and 4 out 24 responded to cit-LL37 (17%)]. As shown in **Supplementary Figure S5**, we plotted SI values of proliferation to native LL37 versus SI of proliferation to cit-LL37 or carb-LL37, and SI for cit-LL37 versus carb-LL37 SI. The results identified the responses to LL37 and carb-LL37 (for both CD4, **Supplementary Figures S5a–c** and CD8 T-cell responses, **Supplementary Figure S5d**) as the most significant correlations (Pearson’s test). For CD4 T cells only, a significant correlation was present for responses to cit-LL37 and the native antigen or for cit-LL37 and carb-LL37 responses. Altogether, these results confirm that LL37 is a strong autoantigen for psoriasis T cells (17) and reveal that the LL37-modified versions are also immunogenic. Moreover, the correlation analyses indicate a multi-specific response in each patient, although we cannot rule out the possibility that, especially for LL37- and carb-LL37-directed T-cell responses, cross-reactivity is present.

### 3.3 T-cell responses to carb-LL37 correlate with disease activity in psoriasis

**Figure 5:**
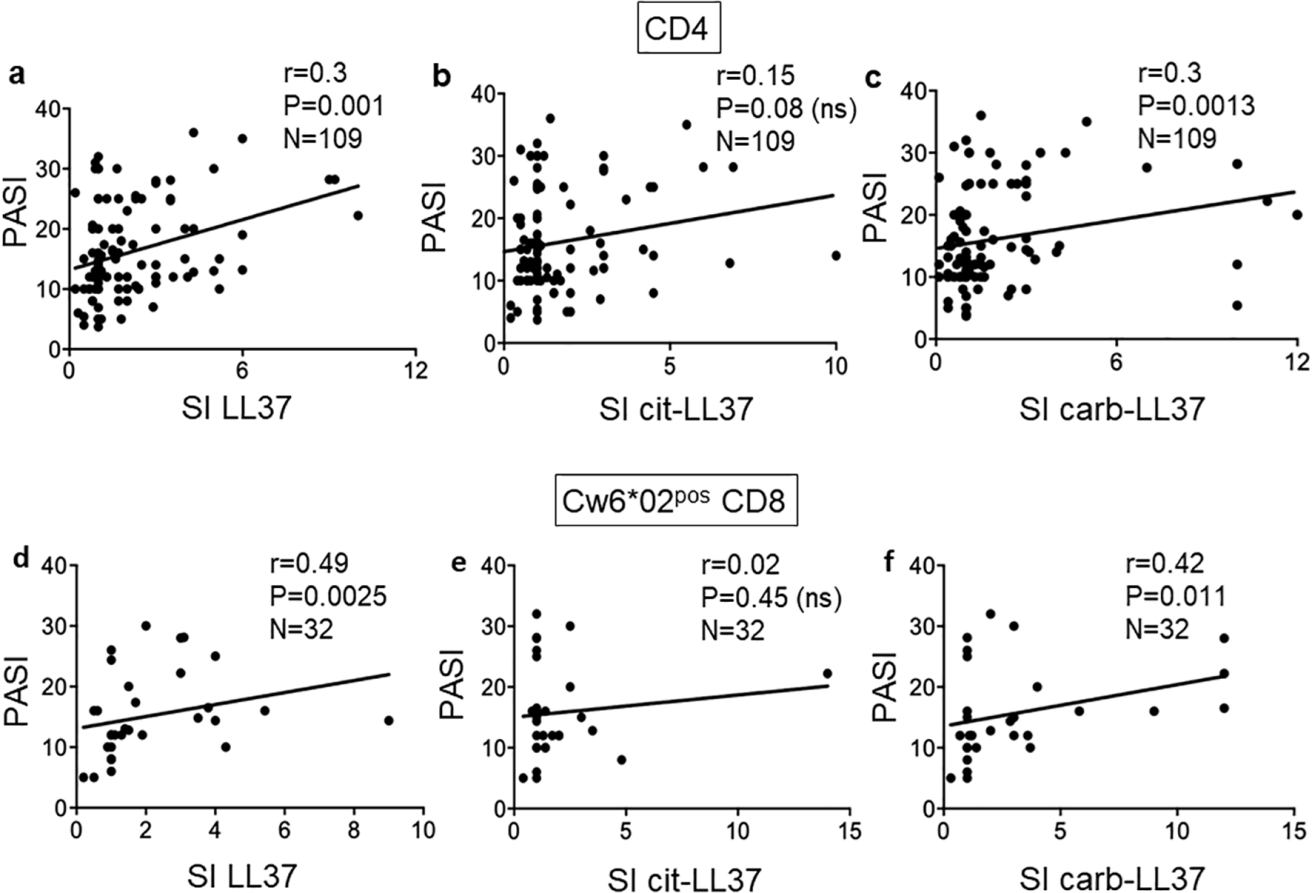
T-cell proliferation to carb-LL37 correlates with PASI. Pearson’s correlation was used to assess the correlation between CD4 T-cell responses to LL37 and modified LL37 and disease activity, expressed as SI and PASI, respectively **(a–c)**. **(d–f)** Correlation between CD8 T-cell responses to native LL37, cit-LL37, and carb-LL37, respectively, in Cw6*02-positive patients with psoriasis. Pearson’s correlation coefficient “*r*,” significance “*p*,” and sample size “*N*” are reported on each graph.

We next plotted the results of T-cell proliferation indexes against the disease activity measured as PASI (**Figure 5**). Notably, the proliferative response to carb-LL37 (**Figure 5c**), unlike the response to cit-LL37 (**Figure 5b**), significantly correlated with PASI. CD4 T-cell proliferation to native LL37 correlated with PASI, as previously reported (**Figure 5a**), confirming previous observations (17). The data may suggest a major involvement of the carbamylation, as compared to the citrullination pathway, in psoriasis, which seems in line with results obtained in PsA (18). Regarding the CD8 T-cell proliferative responses, no correlation was evident in the entire group of 82 patients (LL37: *r* = 0.00, *p* = 0.04, *N* = 82; cit-LL37: *r* = 0.08, *p* = 0.27, *N* = 82; carb-LL37: *r* = 0.11, *p* = 0.2, *N* = 82); therefore, we sorted the patients into two groups, a Cw6*02-positive and a Cw6*02-negative group. CD8 T-cell responses to native LL37 and carb-LL37 correlated with PASI in the Cw6*02-positive patients (*N* = 32) (**Figures 5d, f**). Responses to cit-LL37 did not correlate with PASI in the Cw6*02-positive patients (**Figure 5e**). These data suggest that in Cw6*02-positive patients, activation of CD8 T cells may have a pathogenic significance, as Cw6*02 is an HLA-Class I molecule that presents several LL37 epitopes to CD8 T cells (17). When we considered the 24 patients with psoriasis affected by PsA (see **Supplementary Table S1**), a correlation between PASI and CD4 T-cell responses to LL37, cit-LL37, and carb-LL37 was evident for all three antigens (native LL37: *r* = 0.41, *p* = 0.02, *N* = 24; cit-LL37: *r* = 0.40, *p* = 0.023, *N* = 24; carb-LL37: *r* = 0.48, *p* = 0.009, *N* = 24). These results suggest that not only LL37, but also its modified forms, could have a pathogenic significance in psoriasis and PsA (17, 18).

### 3.4 Cit-LL37- and carb-LL37-specific T cells display a different T-helper phenotype

**Figure 6:**
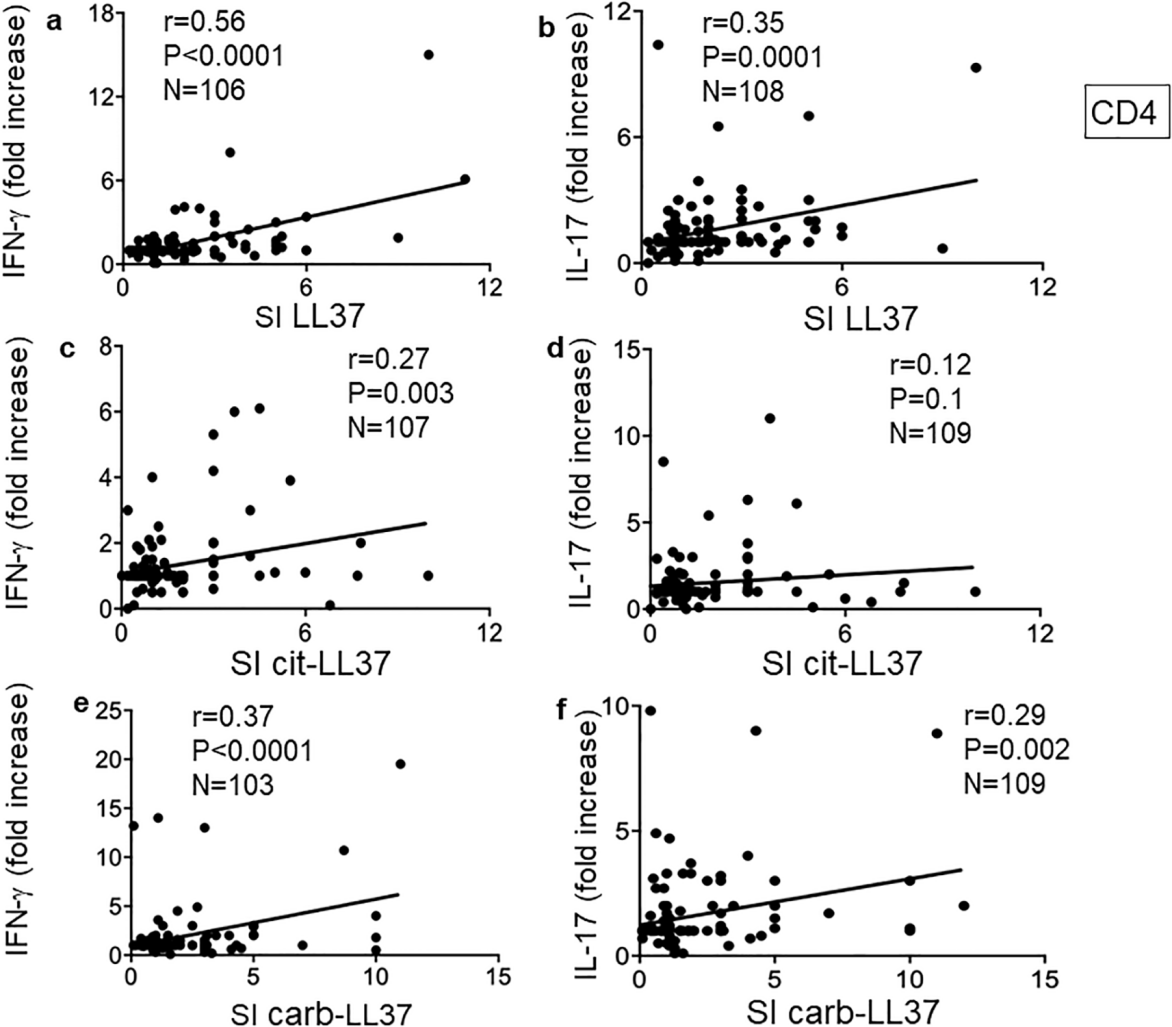
Carb-LL37, like LL37-specific CD4 T cells, are mainly Th1 and/or Th17 cells. **(a, c, e)** IFN-γ and **(b, d, f)** IL-17 were measured by intracellular staining in psoriasis CD4 T cells proliferating to native LL37 **(a, b)**, cit-LL37 **(c, d)**, and carb-LL37 **(e, f)**. Values of fold increase of IFN-γ or IL-17 with respect to responses to control antigen (REV peptides, see Materials and Methods) were plotted against SI for proliferation to the same antigens. Pearson’s correlation coefficient “*r*,” significance “*p*,” and sample size “*N*” are reported on each graph.

We previously demonstrated that autoreactive CD4 T cells specific for LL37 had mainly a profile of Th1 and/or Th17 cells (17). Thus, we analyzed cytokine profiles of the CD4 T cells responding to the native or modified LL37 in our cohort. An example of flow cytometry analysis of cytokines is depicted in **Supplementary Figure S6**. We expressed cytokine increase in response to LL37 and modified LL37 with respect to control peptides (REV) (fold increase, see Materials and Methods), and we used these values to make a correlation analysis. **Figure 6** reports results for IFN-γ and IL-17 expression: here, we addressed the coupling of CD4 T-cell proliferation with these two cytokines. To do so, we performed correlation assays between SI for proliferation and fold increase of cytokine-positive CD4 T cells in the same assays.

Pearson’s correlation analysis showed that CD4 T-cell proliferation (SI) correlated with IFN-γ in all three conditions (CD4 T cells responding to LL37, cit-LL37, and carb-LL37), although correlation was the highest for native LL37-specific CD4 T-cell responses (**Figure 6a**) and the lowest for cit-LL37 specific CD4 T-cell responses (**Figure 6c**). Pearson’s analysis also showed that LL37- and carb-LL37-specific CD4 T-cell proliferation (**Figures 6b, f**), unlike proliferation to cit-LL37 (**Figure 6d**), correlated with an increase of IL-17-producing CD4 T cells. This result suggests that T cells specific for carb-LL37, like cells specific for the native antigen, belong to a Th1/Th17 subset. In case of responses to cit-LL37, CD4 T cells less often produced these two cytokines.

For the 24 patients with PsA, the correlations between proliferation and IFN-γ were the following: for LL37: *r* = 0.41, *p* = 0.03, *N* = 24; for cit-LL37, *r* = 0.45, *p* = 0.017, *N* = 24; for carb-LL37: *r* = 0.64, *p* = 0.0007, *N* = 24. For IL-17 production, the correlations were the following: *r* = 0.38, *p* = 0.034, *N* = 24 for LL37; *r* = 0.58, *p* = 0.0015, *N* = 24 for cit-LL37; and *r* = 0.8, *p* < 0.0001, *N* = 24 for carb-LL37. Thus, for patients with PsA, the IL-17 response of CD4 T cells also correlated with proliferation. Altogether, these results highlight qualitative differences among LL37-, cit-LL37-, and carb-LL37-specific CD4 T cells, in patients with psoriasis.

### 3.5 Cit-LL37-specific T cells show a more oriented T-helper follicular-like phenotype

**Figure 7:**
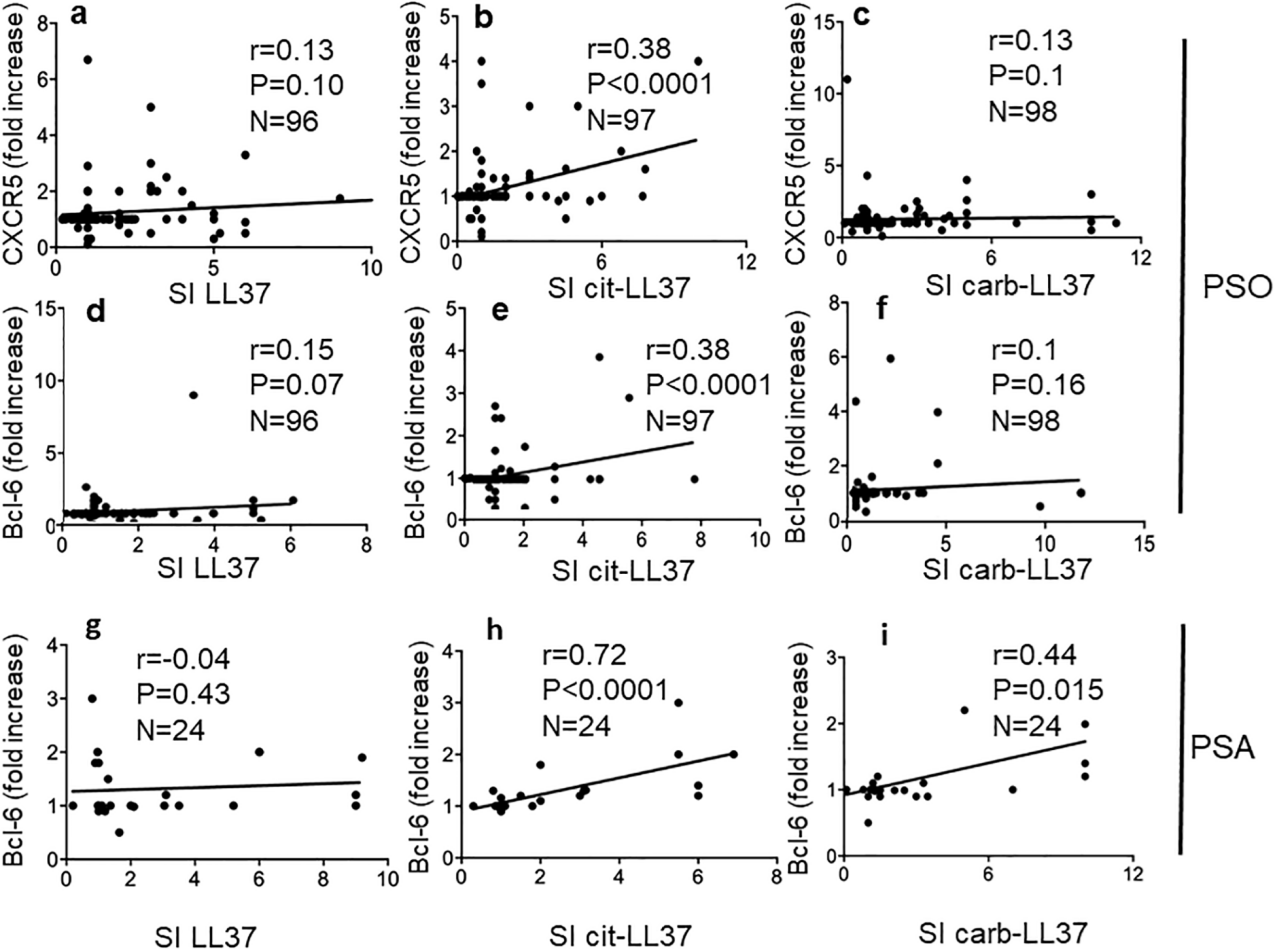
Cit-LL37-specific CD4 T cells significantly upregulate Thf-like markers. CXCR5 and Bcl-6 were measured by membrane (after 5 days of culture, proliferation assays) and intracellular staining (after 48 h of culture, see Materials and Methods), respectively, and assessed by flow cytometry, in psoriasis CD4 T cells proliferating to native LL37 **(a, d, g)**, cit-LL37 **(b, e, h)**, or carb-LL37 **(c, f, i)**. Fold increase values of each marker expression were plotted against SI for proliferation to the same antigens, to make a correlation using Pearson’s correlation test **(a–f)**. For correlations in **(g–i)**, calculated in the group of 24 patients with psoriasis with PsA, we used Spearman’s correlations test, due to lower sample size. Correlation coefficient “*r*,” significance “*p*,” and sample size “*N*” are reported on each graph.

**Figure 8:**
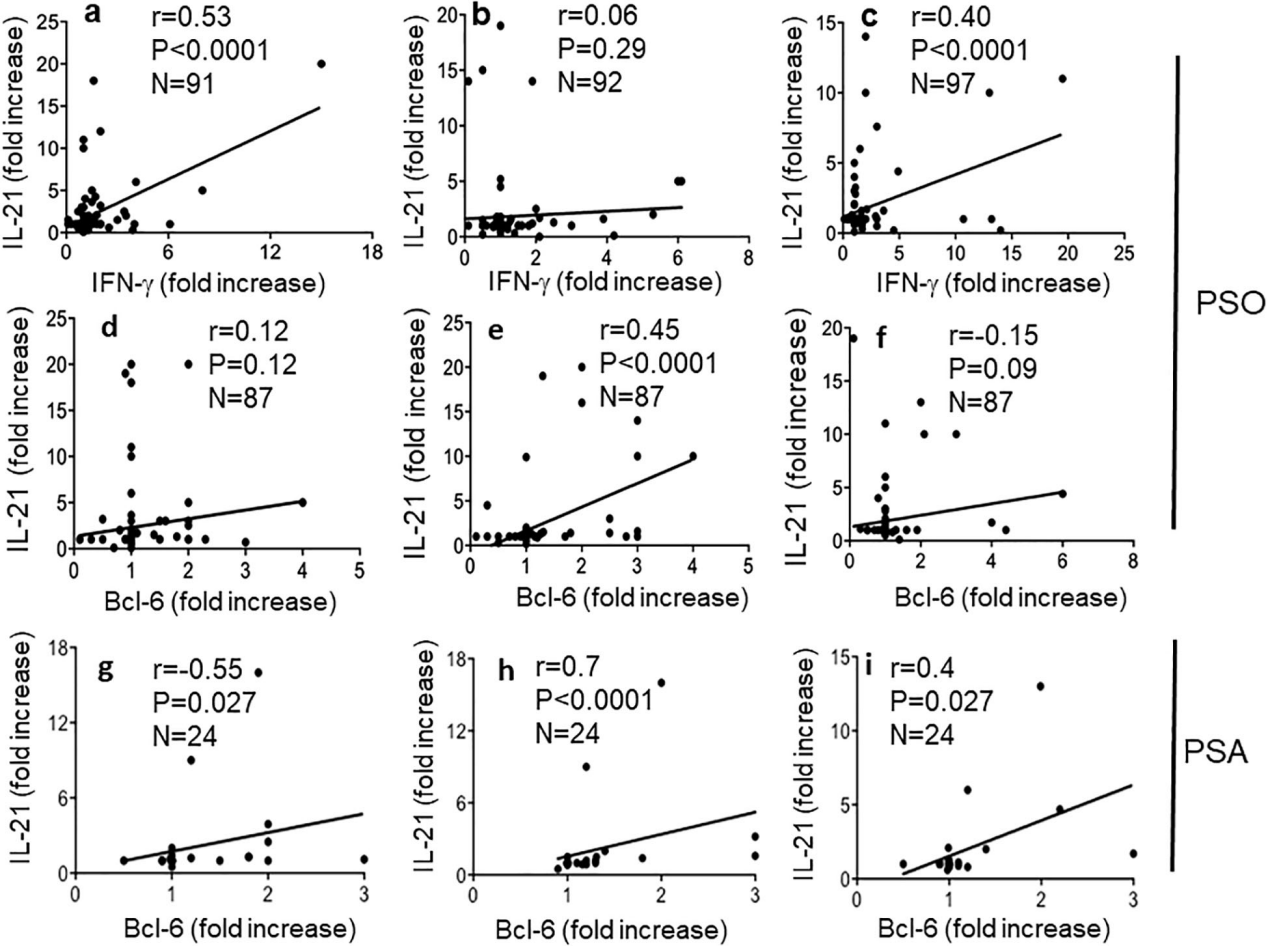
Cit-LL37-specific CD4 T cells produce IL-21 concomitant with Bcl-6 induction. IL-21, IFN-γ (in proliferation assays), and Bcl-6 increase (in 48-h culture assay, see Materials and Methods), in response to antigen stimulation, were assessed by intracellular staining and flow cytometry, in proliferating CD4 T cells, as in **Figure 7**. We correlated **(a–c)** the increase of IFN-γ and IL-21 in CD4 T cells responding to native LL37 **(a)**, cit-LL37 **(b)**, and carb-LL37 **(c)** using Pearson’s correlation assay. In **(d–f)** and **(g–i)**, we correlated the increase of IL-21-positive cells gated on proliferating CD4 T cells with the Bcl-6 fold increase, in response to native LL37 **(d, g)**, cit-LL37 **(e, h)**, and carb-LL37 **(f, i)**. In **(d–f)**, we used Pearson’s correlation tests, whereas in **(g–I)**, we used Spearman’s correlation test due to lower sample size. Correlation coefficient “*r*,” significance “*p*,” and sample size “*N*” are reported on each graph.

To address further differences among the native LL37- and modified LL37-specific CD4 T cells, we analyzed T-helper follicular (Thf) cells, which can be altered in psoriasis according to a prior study (36). In our previous study, we found that LL37-specific CD4 T cells did not usually upregulate CXCR5, one of the markers of Thf cells (17). Since CXCR5 itself does not definitely identify Thf cells (37), we measured CXCR5 and Bcl-6 upregulation in the gated Ki67^+^CD3^+^CD4^+^ cells (LL37 and modified LL37-specific CD4 T cells), responding to the native and modified LL37 (see **Supplementary Figure S7** for gating strategy for Bcl-6 expression, and **Supplementary Figure S8** for gating strategy of CXCR5 positivity in the same gated population). The data show that CXCR5 and Bcl-6 can be upregulated in LL37 and modified LL37-proliferating cells. However, fold increase of both CXCR5 and Bcl-6 correlated with proliferation indexes (SI) only for cit-LL37-specific CD4 T cells (**Figures 7b, e**), but not for the native LL37- and carb-LL37-specific CD4 T cells (**Figures 7a, c, d, f**). These results further indicate that the polarization of cit-LL37 specific T cells may be different from that of both native LL37- and carb-LL37-specific T cells. In the subgroup of 24 patients with PsA, a correlation between Bcl-6 fold increase and CD4 T-cell proliferation was the highest in the case of CD4 T cells responding to cit-LL37 (**Figure 7h**), as compared to the cells responding to native LL37 (no correlation) or carb-LL37 (**Figures 7g, i**), respectively. Since Th1, Th17, and Thf cells can all produce IL-21, which plays a role in psoriasis (38), we analyzed IL-21 in our proliferation assays. **Supplementary Figure S9** shows the gating strategy for assessing IL-21-positive CD4 T cells. Similar to the determination of IFN-γ or IL-17 positivity of **Supplementary Figure S6**, expression of IL-21 was quantified as single staining in Ki67^+^ CD3^+^CD4^+^ cells (SI was calculated in the same way as for IL-17 and IFN-γ). Stimulation with LL37 and modified LL37 could induce IL-21 upregulation in CD4 T cells, as compared to cells stimulated with the control antigens. Correlation analyses indicated that production of IL-21 (expressed as SI) correlated positively with the production of IFN-γ for CD4 T cells stimulated with native LL37 and carb-LL37 (**Figures 8a, c**). This suggests that CD4 T cells producing IL-21 may belong to Th1 type of cells in these two groups. In contrast, IL-21 induction did not significantly correlate with IFN-γ production in the case of responses to cit-LL37 (**Figure 8b**). This might suggest that the IL-21-producing cells in the CD4 T-cell pool responding to cit-LL37 were not all Th1 cells. Indeed, IL-21 and Bcl-6 increase correlated positively only in cit-LL37-responder CD4 T cells (**Figure 8e**) and not in T cells stimulated by the native or carb-LL37 (**Figures 8d, f**). This result might suggest that CD4 T cells that upregulate IL-21 in the cit-LL37 responder group are those that can also upregulate Bcl-6. We do not have a direct co-staining as the number of cells expressing Bcl-6 in the Ki67^+^ T-cell pool was a limiting factor. In the PsA group, a correlation between upregulation of Bcl-6 and IL-21 was the highest in the cit-LL37 responding CD4 T cells (**Figure 8h**), as compared to the native LL37 or carb-LL37 (**Figures 8g, i**).

### 3.6 Patients with psoriasis show occasional antibody reactivity to LL37/modified LL37, which does not correlate with T-cell proliferation and PASI

Since we have previously shown that patients with psoriasis can occasionally develop anti-LL37, anti-cit-LL37, or anti-carb-LL37 antibodies (18), we measured the same antibody specificities in our cohort. Twelve (11%), 17 (16%), and 6 (5%) out of the 109 patients had antibody responses to LL37, cit-LL37, and carb-LL37 (**Supplementary Figure S10a**), respectively, as compared to HD. To address whether the antibody reactivity was truly directed to the native LL37, cit-LL37, and carb-LL37 and not merely induced by the presence of the amino acids citrulline and homocitrulline themselves, the psoriasis sera that were positive for such antibody responses were tested in parallel for reactivity to control peptides (REV), either in the native or in the modified form (**Supplementary Figure S10b**). The response of each serum to LL37, cit-LL37, and carb-LL37 was significantly higher as compared to the response to the control REV peptides, indicating specificity of the responses for the sequence of LL37, cit-LL37, or carb-LL37 and not merely for citrullines and homocitrullines themselves. No correlations were present between capacity to respond to LL37 or modified LL37 (CD4 T-cell proliferation) and antibody reactivity (**Table 1**). This correlation was not evident for the 24 patients with PsA either. Of note and surprisingly, the correlation between CD4 T-cell proliferation to cit-LL37 and antibody responses to carb-LL37 was negative *r* = −0.38, *p* = 0.033, *N* = 24 (all correlations are reported in **Table 1**). Regarding the correlation between anti-LL37 antibody response and PASI, no significant correlation was detectable in all patients with psoriasis and in the subgroup of patients with psoriasis with PsA as reported in **Table 2**. Criss-cross correlations between antibody reactivity to native LL37, cit-LL37, or carb-LL37 were high (LL37 OD vs. cit-LL37 OD: *r* = 0.8, *p* < 0.0001, *N* = 109; LL37 OD vs. carb-LL37 OD: *r* = 0.71, *p* < 0.0001, *N* = 109; cit-LL37 OD vs. carb-LL37 OD: *r* = 0.75, *p* < 0.0001, *N* = 109), which may suggest a consistent multi-specificity of the antibody response, and perhaps cross-reactivity. Overall, the data show that the response to LL37 and modified LL37 was infrequent in psoriasis, and it was not possible to identify a correlation of this humoral response with PASI or T-cell proliferation in the patients with psoriasis, and not even for those patients with psoriasis associated to PsA.

**Table 1:** No correlation between antibody responses and T-cell responses.

PSO (109)	Anti-LL37 CD4 T-cells	Anti-cit-LL37 CD4 T-cells	Anti-carb LL37 CD4 T-cells
**Antibody reactivity to LL37**	*r=0.15, P=0.06* *N=109*	*r=0.05, P=0.3* *N=109*	*r=0.12, P=0.1* *N=109*
**Antibody reactivity to cit-LL37**	*r=-0.06, P=0.25* *N=109*	*r=0.09, P=0.15* *N=109-*	*r=-0.07, P=0.22* *N=109*
**Antibody reactivity to carb-LL37**	*r=0.12, P=0.1* *N=109*	*r=0.00, P=0.5* *N=109*	*r=0.11, P=0.11* *N=109*
PSO with PSA (24)	Anti-LL37 CD4 T-cells	Anti-cit-LL37 CD4 T-cells	Anti-carb LL37 CD4 T-cells
**Antibody reactivity to LL37**	*r=0.02, P=0.45* *N=24*	*r=0.06, P=0.38* *N=24*	*r=0.12, P=0.10* *N=109*
**Antibody reactivity to cit-LL37**	*r=-0.29, P=0.081* *N=24*	*r=-0.24, P=0.12* *N=24-*	*r=-0.18, P=0.19* *N=24*
**Antibody reactivity to carb-LL37**	*r=-0.33, P=0.06* *N=24*	***r=-0.38, P=0.033*** ***N=24***	*r=-0.3, P=0.07* *N=24*

Correlation between antibody responses to native and modified LL37 is reported for all patients and for the subgroup of 24 patients with psoriasis and PsA. Correlation for the entire cohort was performed with Pearson’s test and that for the 24 patients with psoriasis with PsA was performed with Spearman’s correlation test. The bold value indicates statistically significant correlation.

**Table 2:** No correlation between antibody responses to native and modified LL37 and PASI.

PSO (109)	PASI
**Antibody reactivity to LL37**	*r=-0,08, P=0.19* *N=109*
**Antibody reactivity to cit-LL37**	*r=-0.08, P=0.22* *N=109*
**Antibody reactivity to carb-LL37**	*r=0.07, P=0.1* *N=109*

PSO with PSA (24)	PASI
**Antibody reactivity to LL37**	*r=0.00, P=0.49* *N=24*
**Antibody reactivity to cit-LL37**	*r=-0.12, P=0.28* *N=24*
**Antibody reactivity to carb-LL37**	*r=-0.15, P=0.47* *N=24*

Correlation for the entire cohort was performed with Pearson’s test and that for the 24 patients with psoriasis with PsA was performed with Spearman’s correlation test.

### 3.7 IFN-I concentration increases IL-21 expression but not Thf markers

**Figure 9:**
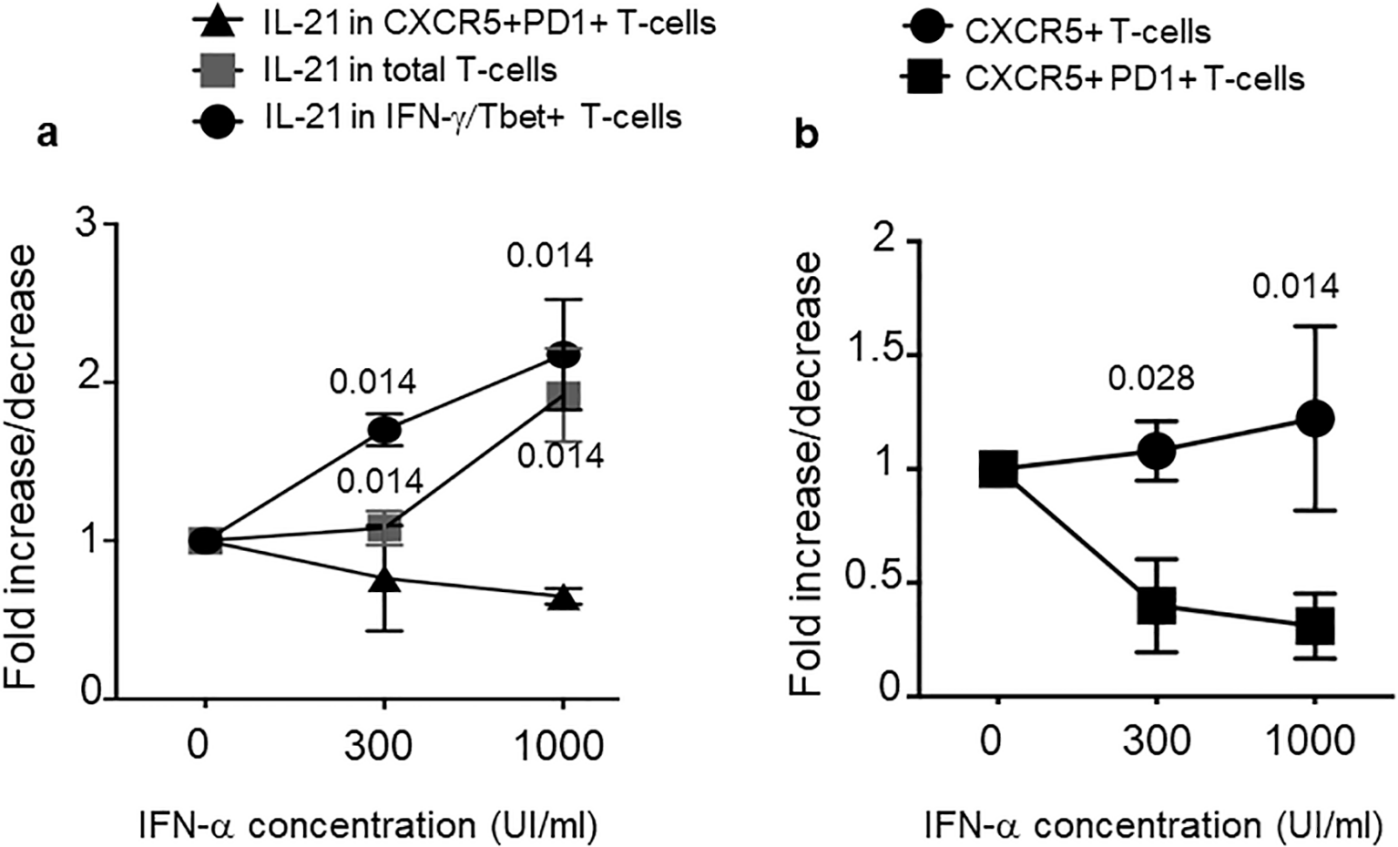
IFN-α concentration increases IL-21 but not Thf markers. MDDC were co-cultured with T cells of patients with psoriasis for 6 days in the absence (0) or presence of two different concentrations of IFN-α (300 UI/mL and 1,000 UI/mL). T cells were washed and stained for markers of Th1 or Thf cells and, by intracellular staining, for IL-21 and IFN-γ. We gated on CD4 T cells that were Ki67^+^ (proliferating cells). **(a)** Fold increase/decrease of IL-21 producing CD4 T cells with respect to cell phenotype (as indicated). **(b)** Fold increase/decrease of CD4 T cells expressing CXCR5 alone or expressing both CXCR5 and PD1 (as indicated), in response to two different doses of IFN-α. Graphs are representative of three different experiments performed in duplicate from three different donors. *p*-values are from Student’s *t*-test. Vertical bars are standard errors of the mean.

In an attempt to understand why psoriasis cit-LL37-specific T cells appeared more skewed towards a Thf-like phenotype, we set up an *in vitro* model system of T-cell activation. The working hypothesis came from the observations that IFN-I affects T-helper cell polarization in favor of a Th1 response (39). In contrast, conflicting results are present regarding the IFN-I ability to favor Thf polarization (40). At one end, IFN-I favors IL-21 production (41); at the other end, IFN-I has been shown to refrain Thf polarization in favor of a Th1 polarization and not to induce directly IL-21 (40, 42). We reasoned that the different interferogenic properties of LL37 and modified LL37 might thus affect the polarization of LL37- or modified LL37-specific psoriasis T cells. Indeed, we have previously shown that only native LL37 and carb-LL37 (although the latter at a lower extent as compared to the native antigen) promoted the IFN-α production by pDCs (22). We reasoned that a way to assess the role of IFN-α in T-cell polarization was to mimic the *in vivo* T-cell activation occurring in psoriasis T cells in a milieu of high or low IFN-α. Therefore, we cultured psoriasis T cells with MDDCs (26, 27, 43), as typical alloantigen-presenting cells, in the presence of high (1,000 U/mL) or low (300 U/mL) human recombinant (hr) IFN-α concentration, administered at the time of the first alloantigen encounter (**Figure 9a**). After 6 days of culture, we performed an intracellular staining of proliferating (Ki67^+^) T cells to address Thf and Th1 marker expression, as well as cytokine production. The results showed that total IL-21^+^ T cells tended to increase in the presence of growing concentration of IFN-α into the cell cultures (**Figure 9a**). However, IL-21 increased in Tbet^+^ T cells (Th1), but not in CXCR5^+^PD1^+^CD4-T cells (Thf-like cells). Considering the surface phenotype of the responder CD4 T cells, it appeared that the number of CXCR5^+^CD4 T cells remained mostly stable, independently from the IFN-I dose, whereas the CXCR5^+^PD1^+^CD4 T cells decreased (**Figure 9b**). These data suggest that IFN-α concentration (which is likely tuned by local expression of LL37, cit-LL37, and carb-LL37) may be a significant factor in the polarization of responder CD4 T cells, with a preferential skew towards a Th1 phenotype in the presence of high IFN-α concentration.

### 3.8 LL37 and modified LL37 differently stimulate antigen-presenting cells and T-helper cell polarization

**Figure 10:**
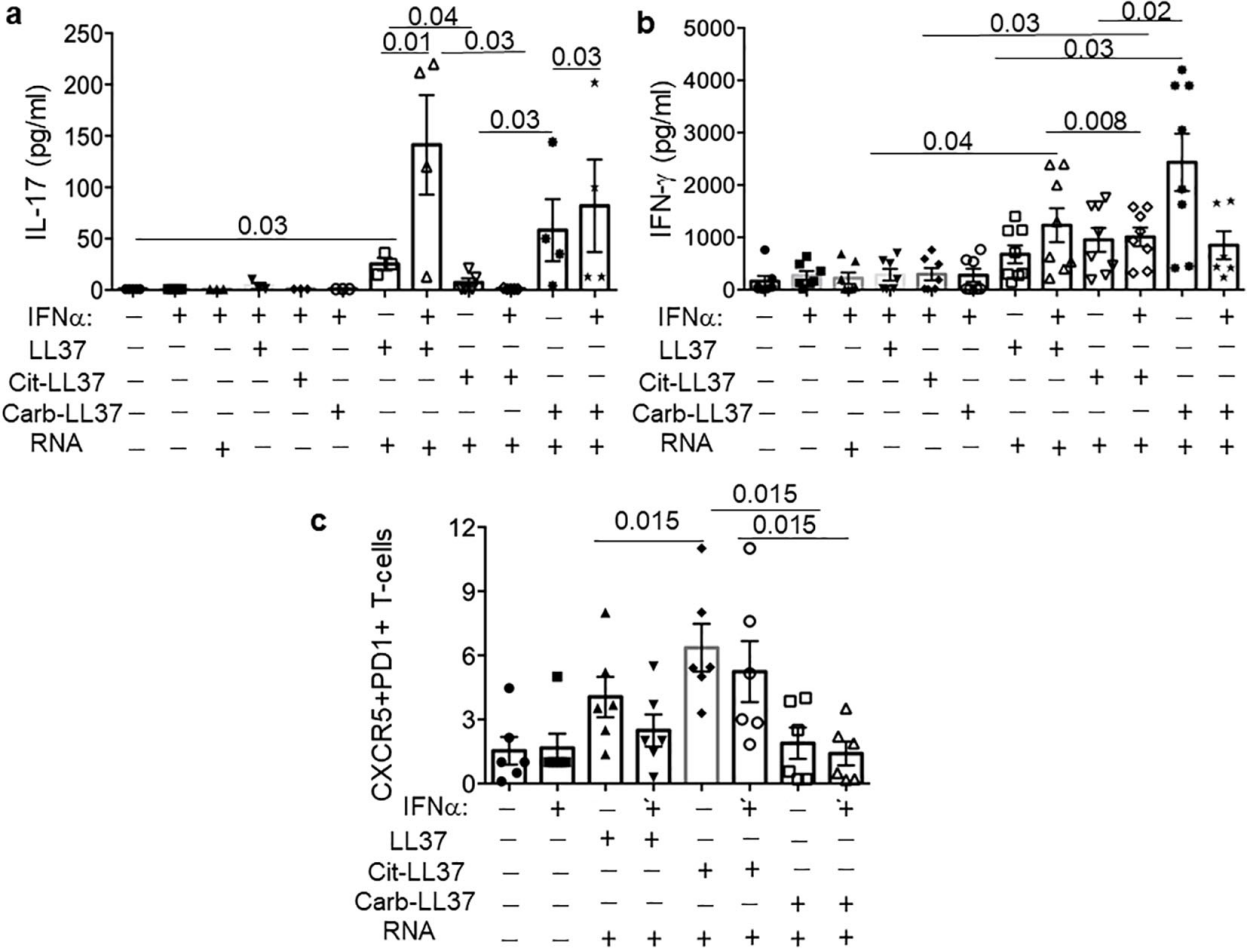
MDDC treated with LL37 and modified LL37 in complex with human RNA have different T-helper polarizing capacities. MDDCs were treated with a mixture of LL37, cit-LL37, and carb-LL37 and human RNA or with LL37, cit-LL37, and carb-LL37 alone, for 24 h, and then co-cultured with allogeneic responder CD4 T cells (isolated as reported in Materials and Methods). After 6 days, the cytokines IL-17 and IFN-γ were assessed by ELISA **(a, b)** in the culture supernatants; CXCR5 and PD1 markers were measured in the CD T cells that were Ki67^+^, by flow cytometry in the culture conditions indicated in **(c)**. See **Supplementary Figure S12** for gating strategy. Reported are cumulative data of six different experiments performed with MDDCs of different donors. Horizontal bars are the means, vertical bars are the standard errors of the mean, and the *p*-values are from Wilcoxon’s test.

To set up a T-cell stimulation assay, which could more precisely mirror the psoriasis skin environment, we used an additional culture system. Here, we stimulated MDDCs with LL37–RNA, cit-LL37–RNA, and carb-LL37–RNA mixtures (or the peptides alone), to mimic the effect that such peptides could have *in vivo* on antigen-presenting cells (APCs), namely, myeloid DCs, in an inflammatory context. These assays were also performed in the presence/absence of IFN-α (1,000 U/mL), to mimic the effect that native LL37 and modified LL37 could have in the microenvironment where T-cell activation by LL37 or modified LL37 occurs (and where pDCs are present (44),). The MDDC culture supernatants, analyzed after 24-h stimulation by ELISA, revealed that MDDCs were indeed stimulated by LL37–RNA, and carb-LL37–RNA, but not by cit-LL37–RNA. Indeed, IL-12 and TNF-α (Th1-polarizing cytokines) and IL-23 (a Th17-polarizing factor) were all detected in the culture medium of MDDCs in response to LL37 and RNA or carb-LL37 and RNA stimulation (IFN-α treatment further increased these cytokines) (**Supplementary Figure S11**). The analysis of the culture supernatants of the T-cell polarization experiments (cumulative data in **Figures 10a, b**) evidenced a release of IL-17 in the presence of native and carb-LL37 (**Figure 10a**) and RNA conditioned MDDCs, but not in the presence of cit-LL37 and RNA conditioned MDDCs. Moreover, the IFN-γ production was significantly higher in LL37–RNA pre-conditioning cultures when IFN-α was present (**Figure 10b**). Flow cytometry analysis of the CD4-T cells after 1 week of culture with MDDCs treated in the culture conditions of **Figure 10c** also showed that CXCR5^+^PD1^+^CD4 T cells were higher in the cit-LL37–RNA culture condition (see **Supplementary Figure S12** for gating strategy). In the conditions where LL37 and carb-LL37 were added in complex with RNA (either in the presence or in the absence of IFN-α), these CXCR5^+^PD1^+^CD4 T cells appeared less expanded. These results suggest that, *in vitro*, LL37 and modified LL37 can have different T-helper cell polarization capabilities.

## 4 Discussion

In this study, we provide evidence that both cit-LL37 and carb-LL37 are present in the psoriasis lesional skin, at skin sites where neutrophils are present, as shown by the presence of MPO staining in the psoriasis skin biopsies. NETosis was also detected at the same sites. T cells of patients with psoriasis respond to both native and modified LL37. Thus, response to the modified autoantigen LL37 is very likely to be in correlation with a neutrophil infiltrate.

Indeed, both citrullination and carbamylation are irreversible PTMs of cationic molecules rich in arginines or lysine (or leucines), which are favored in the presence of a neutrophil-driven inflammation. Citrullination, an enzymatic reaction due to peptidylarginine deiminase (PAD) enzymes, can affect several self-proteins. It can occur directly as the results of NETosis, but can be also favored by the release of PADs during neutrophil degranulation (24, 25, 35, 45). The more or less prominent presence of citrullination can also depend on the type of NETosis induced (46, 47). Carbamylation is a non-enzymatic reaction. However, presence in the skin of MPO could be crucial, as MPO uses hydrogen peroxide (H_2_O_2_) to oxidize thiocyanate (SCN^−^), forming isocyanic acid (HOCN). HOCN is highly reactive and modifies lysine residues on proteins, leading to the formation of carbamylated proteins (30–32). MPO can be released during NETosis, together with PADs, but can be also released via degranulation, independently from NETosis induction (34). Thus, a neutrophilic infiltrate could, by itself, already explain the carbamylation of LL37. In addition, signs of NETosis were visible in the psoriasis skin lesions in our LSCM experiments, while NET-related MPO–DNA complexes, correlating moderately with the PASI, were present in the blood of patients with psoriasis. In recent years, several studies have suggested that NETosis occurs in psoriasis skin as well as in the PsA joints, and indicated NET formation in the skin to be a relevant phenomenon for inflammation (48–53). Our analyses of psoriasis skin biopsies and circulating MPO–DNA complexes are in agreement with these reports. In addition, our data indicate a role for neutrophils and, likely, NETosis in determining the quantity (T-cell activation) and quality (T-helper polarization) of the adaptive immune response to native and modified autoantigens, which could play a role in the rupture of tolerance. The location (the dermis) of cit-LL37 or carb-LL37 was consistent with all these assumptions, as it indicates that these peptides are not directly produced by keratinocytes, but it is the inflammatory milieu, where neutrophils accumulate, that is the most likely determinant of these peptide modifications. Notably, we previously demonstrated that patients with psoriasis with PsA harbored CD4 T cells and antibodies responding to cit-LL37 and, especially, to carb-LL37 (18). In PsA, we also detected an antibody response to cit-LL37 and carb-LL37, which correlated with several disease parameters (18). Occasional autoantibodies to LL37 were also detectable in patients with psoriasis without PsA, but the significance of this observation remained elusive (18). Here, the data still fail to illuminate a relationship between anti-LL37 antibodies and disease activity (PASI) in patients with psoriasis, as such antibodies are infrequent.

Correlation analyses indicate that T-cell responses to native LL37, cit-LL37, and carb-LL37 are multi-specific and suggest the existence of cross-reactive responses. We can only hypothesize this cross-reactivity, as we did not clone T cells from patients with psoriasis. However, since correlations between responses to native LL37 and carb-LL37 were the highest, and LL37- and carb-LL37-specific CD4 T cells share a common T-helper phenotype, it is possible to speculate that carb-LL37-specific T cells cross-react with the native antigen. This further reinforces the assumption that carbamylation, in particular, may play a role in the rupture of T-cell tolerance in psoriasis. Indeed, the magnitude of the carb-LL37-specific T-cell response (in terms of proliferative capacity) correlates with PASI (17), whereas a similar correlation was not observed in the case of responses to cit-LL37.

**Figure 11:**
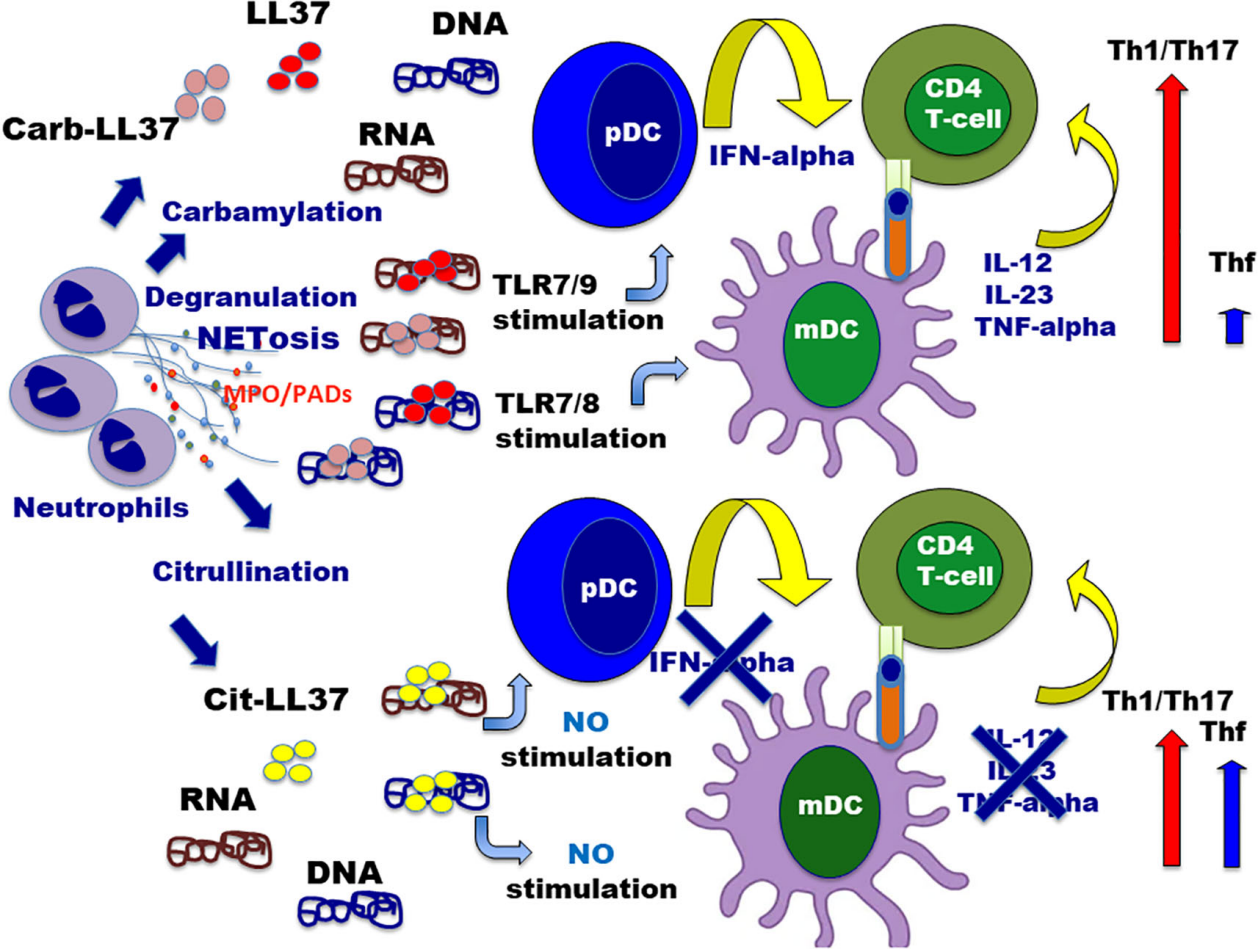
Interplay between neutrophil infiltrate and activation/polarization of autoreactive CD4 T cells specific for LL37 and modified LL37. The cartoon summarizes the relationship between neutrophil activity (activation with degranulation and/or NET extrusion), with the release of PADs or MPO, and the polarization of autoreactive T-helper cells in psoriasis. Neutrophils are supposed to be activated and undergo NETosis via multiple possible stimuli such as inflammatory cytokine stimulation (IFN-I, TNF, and IL-8) or via TLRs triggering (49–51, 53). Although not represented in the picture, LL37–RNA may be “pre-formed” complexes extruded by NETting neutrophils, which could induce, in turn, TLR8 triggering in neutrophils (50, 51), with consequent NETosis amplification via the implementation of a vicious cycle. Once neutrophils degranulate or extrude NET, LL37–DNA or LL37–RNA complexes are formed (which could also contain mitochondrial DNA (60)). The action of MPO favors carbamylation of self-proteins (including LL37), whereas cit-LL37 can be produced by released PADs enzymes or directly before NETosis by the activation of PADs enzymes by Ca^++^ influx (46, 61). Depending on the respective amounts of cit-LL37 and carb-LL37 (maybe also linked to the type of NETosis induced), in the milieu of inflammation, APC can be conditioned to amplify a prevalent Th1/Th17 response (LL37–DNA/RNA or carb-LL37–DNA/RNA complexes) or allow a larger amplification of self-reactive Thf-like cells (due to LL37 citrullination, which abrogates the “adjuvant-like activity of LL37).

In a previous study, we have shown that native LL37, cit-LL37, and carb-LL37 have different interferogenic abilities when complexed with self-DNA (22). Knowing that LL37–RNA complexes stimulate myeloid DC (MDDC) via TLR7/8 (54), we repeated here these experiments using the same cell culture system of MDDC to demonstrate that both LL37 and carb-LL37 stimulated myeloid DC to secrete Th1/Th17-polarizing factors, whereas cit-LL37 failed to do so. The distinct interferogenic and pro-inflammatory properties of LL37 and modified LL37 may determine the distinct T-cell polarization observed in both *in vitro* models of T-cell activation and *ex vivo* analysis of autoreactive psoriasis T-helper cells. LL37/carb-LL37-specific T cells and cit-LL37-specific T cells appear indeed positioned at two different ends: the former apparently belong to the Th1 and/or the Th17-cell subsets, whereas the cit-LL37-specific T cells appear more Thf-like cell oriented. This reflects the demonstration that LL37 and carb-LL37 have “adjuvant like activity” in complex with nucleic acids, as shown in previous papers (22, 48–51, 54), whereas cit-LL37 does not possess a significant adjuvant-like activity (22, 55). LL37–RNA complexes could be very important in the psoriasis skin. An important question concerns the source of both self-DNA and self-RNA in the psoriasis skin. LL37–DNA complexes could derive from cells dying during inflammation by necrosis (54), although more other studies in psoriasis or other diseases indicate that these complexes are extruded by NETosis (48–51, 55–58). Two interesting papers showed that LL37–RNA complexes might derive mainly from NETting neutrophils (that we identified here in psoriasis skin (50, 51)). Several studies indicate how NET is able to activate immune cells via several TLRs, including DNA or RNA sensing TLRs (48, 50, 51, 53, 56–58). Even more interesting, one of these studies showed that an “RNA–LL37 composite damage-associated molecular pattern (DAMP)” is actually even “pre-stored” in resting neutrophil granules (51). Although we did not search for such LL37–RNA complexes in the skin for technical reasons, it is likely that where NETosis occurs, LL37–RNA complexes will be more massively present. Because of the adjuvant-like activity of LL37, NET has been shown to be linked to IL-17 induction in CD4 T cells (48, 49). This is in keeping with our results. Indeed, carb-LL37 may perform functions similar to the native antigen, according to our *in vitro* data (48, 49). Psoriasis CD4 T cells responding to the native LL37 and even carb-LL37 were indeed the ones that produce more often IL-17 in our analyses. Therefore, our data are in keeping with the previous literature (48, 49, 51). However, we speculate that is the reciprocal expression of cit-LL37 and carb-LL37 (and native LL37) in the tissues, which may determine the polarization of autoreactive T cells in each individual patient. In our *in vitro* model systems of CD4 T-cell polarization presented here, we used recombinant IFN-α to mimic the effect of pDCs *in vitro*, as IFN-α was shown in the past to be crucial for psoriasis plaque formation (44). Also, our *in vitro* experiments of polarization are in keeping with the *ex vivo* analysis of LL37/modified LL37-specific CD4-T cells. In the first model, IFN-α apparently skews the cells towards a Th1 more than to a Thf-like cell phenotype. In the second model, IFN-γ and IL-17 appear mainly induced in T-cell cultures with APC conditioned in the presence of LL37 and carb-LL37, which induce IL-12, IL-23, and TNF-α in MDDC. Thf-like markers were found higher in cit-LL37-polarized CD4 T cells (either in the presence or in the absence of IFN-α). Regarding this latter observation, what it is more likely is that Tfh cells are not directly expanded due to the presence of cit-LL37 and nucleic acids, but are rather the Th1/Th17 cells that are preferentially favored, due to the “adjuvant-like activity” of LL37 and carb-LL37 in complex with self-RNA. Thus, this adjuvant-like effect of LL37 (and carb-LL37) may represent the driving force that expands Th1 and/or Th17 cells at the expenses of the Thf cells (scheme in **Figure 11**). This is of high significance in psoriasis because it explains how both LL37 and carb-LL37 skew the CD4 T-cell response towards a T-helper response that is pathogenic in psoriasis. In contrast, it is possible to hypothesize that a massive citrullination of the autoantigen LL37 could exert a beneficial effect, by suppressing Th1/Th17 cells, which are pathogenic in psoriasis. Citrullination (of LL37) may thus counteract the suppression of Thf cells’ generation induced by IFN-α and the Th1-polarizing cytokines (which are all induced by the native LL37 and carb-LL37). However, a failure to limit an autoreactive Thf response driven by cit-LL37 may generate CD4 T cells that are more prone to provide help for autoantibody production. This is reminiscent of what we have previously observed in SLE (21, 22). In SLE, not only NETosis seems to occur at high levels exposing many autoantigens (56, 59, 60), but phenomena of hypercitrullination of the exposed autoantigen may be at work due to the high complement expression and MAC (membrane attack complexes) deposition (61, 62). This massive citrullination of self-antigens (which also affects LL37) could be responsible for the higher expression of LL37-specific CD4 T cells with a Thf-like phenotype in SLE, which can be instrumental in driving the highly frequent autoantibody response to LL37 and modified LL37 in SLE patients (21, 22). In PsA, autoantibodies to LL37 and modified LL37 are also induced and may play a similar pathogenic effect in the joints (18). Unfortunately, the patients with psoriasis with PsA are low in number in our cohort and the data of a higher expression of these antibodies in patients with PsA is not replicated (18); correlation between cit-LL37 CD4 T-cell responses and antibodies to LL37, cit-LL37, or carb-LL37 was unlikely to be revealed in such a small group.

Our *in vitro* polarization experiments concern CD4 T cells isolated with a method that does not discriminate between naive and memory cells. This can be a limitation. However, LL37-, cit-LL37-, and carb-LL37-specific T cells may derive from memory CD4 T cells that are diverted T-regulatory (Tregs) cells (63). Memory CD4 T cells or Tregs, due to T-cell plasticity, may acquire alternative T-helper properties (64), and the milieu of stimulation can alter their definitive polarization. Unfortunately, a limitation of our data is the lack of a systematic and parallel study of the skin biopsies of each patient, for expression of native and modified LL37, and the concomitant assessment of the corresponding T-cell and antibody responses in the peripheral blood. This is a more complex study to coordinate, as biopsies are not routinely performed on patients with psoriasis.

In conclusion, the evidence that neutrophis are linked to PTMs of the autoantigen LL37, and the detection of NETosis in psoriasis skin shown here and in the abovementioned studies, may indicate that neutrophils are the key cells driving a pathogenic T-cell activation and polarization in psoriasis. What induces NETosis in psoriasis skin is partially understood. NETs can be induced by inflammatory mediators, such as IL-8 or IFN-I or GM-CSF [reviewed in (49, 65, 66)], which are upregulated in psoriasis skin. Stimulation *via* TLR can also induce NET (53, 67). Human neutrophils express several TLRs, with TLR8 being the most expressed single-strand RNA TLR (66). Thus, it is possible to infer that formation of LL37–RNA complexes can induce NET. Indeed, recent papers mentioned above demonstrated that LL37–RNA complexes not only are extruded during NETosis and can fuel inflammation *via* TLR7/8, but also indicate that LL37–RNA complexes act *via* TLR8 on neutrophils to, in turn, induce NETosis; this creates a loop of activation in which NET components further activate NETosis (50, 51). The search of the stimuli that induce NETosis in psoriasis is also important to discriminate the type of NETosis occurring in the skin. Depending on the type of stimulus, more or less citrullination of self-protein could occur, which would alter the balance between the Th1/Th17-driving forces (LL37–DNA/RNA and carb-LL37 DNA/RNA) and the suppression of these forces via citrullination (46, 61, 62). The data reported here can stimulate some considerations among physicians that treat psoriasis. If neutrophils are important in determining the evolution of psoriasis and possibly the rupture of tolerance, as also suggested by the role of carb-LL37 as a psoriasis T-cell antigen, a timely therapy to block neutrophil inflammation and activity could help to reduce autoreactivity. One of the preferred early treatment could be anti-IL-17-blocking agent administration, which limits an exaggerated Th17 activation and, at the same time, inhibits the neutrophil infiltrate into the skin (68, 69). As discussed above, whether early IL-17 block in psoriasis translates, at later stages, into a block of autoreactivity to LL37 and other autoantigens, and to a less frequent PsA development due to lower Thf cell generation, can be only established by larger retrospective and *ad hoc* longitudinal studies in appropriate cohorts of patients with psoriasis. In addition, since evidence of NETosis is present, the use of inhibitors of NETosis could also be a strategy to block psoriasis inflammation, as well as the autoreactivity favored by these inflammatory pathways. *Tofacitinib* (a JAK3/JAK1 inhibitor) regulates neutrophil migration and the formation of NETs and could be useful, especially as an early treatment (70, 71).

## Data Availability

The raw data supporting the conclusions of this article will be made available by the authors, without undue reservation.

## References

[1] Furue M Kadono T. Psoriasis: behind the scenes. J Dermatol. (2016) 43:4–8. doi: 10.1111/1346-8138.13186 26782000

[2] Jacobson CC Kumar S Kimball AB. Latitude and psoriasis prevalence. J Am Acad Dermatol. (2011) 65:870–73. doi: 10.1016/j.jaad.2009.05.047 21920244

[3] Elder JT. Genome-wide association scan yields new insights into the immunopathogenesis of psoriasis. Genes Immun. (2009) 10:201–9. doi: 10.1038/gene.2009.11 PMC2683580 19262574

[4] Deng Y Chang C Lu Q. The inflammatory response in psoriasis: a comprehensive review. Clin Rev Allergy Immunol. (2016) 50:377–89. doi: 10.1007/s12016-016-8535-x 27025861

[5] Ellinghaus D Jostins L Spain SL Cortes A Bethune J Han B. Analysis of five chronic inflammatory diseases identifies 27 new associations and highlights disease-specific patterns at shared loci. Nat Genet. (2016) 48:510–18. doi: 10.1038/ng.3528 PMC4848113 26974007

[6] Elder JT Bruce AT Gudjonsson JE Johnston A Stuart PE Tejasvi T. Molecular dissection of psoriasis: integrating genetics and biology. J Invest Dermatol. (2010) 130:1213–26. doi: 10.1038/jid.2009.319 19812592

[7] Duffin KC Krueger GG. Genetic variations in cytokines and cytokine receptors associated with psoriasis found by genome-wide association. J Invest Dermatol. (2009) 129:827–33. doi: 10.1038/jid.2008.308 18830267

[8] Di Cesare A Di Meglio P Nestle FO. The IL-23/Th17 axis in the immunopathogenesis of psoriasis. J Invest Dermatol. (2009) 129:1339–50. doi: 10.1038/jid.2009.59 19322214

[9] van Baarsen LGM Lebre MC van der Coelen D Snoek BC Gerlag DM Tak PP. IL-17 levels in synovium of patients with rheumatoid arthritis, psoriatic arthritis and osteoarthritis: Target validation in various forms of arthritis. Ann Rheum Dis. (2011) 70:A79. doi: 10.1136/ard.2010.149013.27

[10] Langley RG Elewski BE Lebwohl M Reich K Griffiths CE Papp K. Secukinumab in plaque psoriasis–results of two phase 3 trials. N Engl J Med. (2014) 371:326–38. doi: 10.1056/NEJMoa1314258 25007392

[11] Baeten D Sieper J Braun J Baraliakos X Dougados M Emery P. Secukinumab, an interleukin-17A inhibitor, in ankylosing spondylitis. N Engl J Med. (2015) 373:2534–48. doi: 10.1056/NEJMoa1505066 26699169

[12] Shirley M Scott LJ. Secukinumab: A review in psoriatic arthritis. Drugs. (2016) 76:1135–45. doi: 10.1007/s40265-016-0602-3 27299434

[13] Blair HA Dhillon S. Secukinumab: A review in ankylosing spondylitis. Drugs. (2016) 76:1023–30. doi: 10.1007/s40265-016-0598-8 27255593

[14] Boutet MA Nerviani A Gallo Afflitto G Pitzalis C. Role of the IL-23/IL-17 axis in psoriasis and psoriatic arthritis: the clinical importance of its divergence in skin and joints. Int J Mol Sci. (2018) 19:530. doi: 10.3390/ijms19020530 29425183 PMC5855752

[15] Davidson A Diamond B. Autoimmune diseases. N Engl J Med. (2001) 345:340–50. doi: 10.1056/NEJM200108023450506 11484692

[16] Jin L Wang G. Keratin 17: a critical player in the pathogenesis of psoriasis. Med Res Rev. (2014) 34:438–54. doi: 10.1002/med.21291 23722817

[17] Lande R Botti E Jandus C Dojcinovic D Fanelli G Conrad C. The antimicrobial peptide LL37 is a T-cell autoantigen in psoriasis. Nat Commun. (2014) 5:5621. doi: 10.1038/ncomms6621 25470744

[18] Frasca L Palazzo R Chimenti MS Alivernini S Tolusso B Bui L. Anti-LL37 antibodies are present in psoriatic arthritis (PsA) patients: new biomarkers in psA. Front Immunol. (2018) 9:1936. doi: 10.3389/fimmu.2018.01936 30279686 PMC6154218

[19] Pruijn GJ. Citrullination and carbamylation in the pathophysiology of rheumatoid arthritis. Front Immunol. (2015) 6:192. doi: 10.3389/fimmu.2015.00192 25964785 PMC4410602

[20] Wang Y Liu H Fu Y Kao W Ji Y Liu X. Novel biomarkers containing citrullinated peptides for diagnosis of systemic lupus erythematosus using protein microarrays. Clin Exp Rheumatol. (2019) 37:929–36. 30789148

[21] Lande R Palazzo R Gestermann N Jandus C Falchi M Spadaro F. Native/citrullinated LL37-specific T-cells help autoantibody production in Systemic Lupus Erythematosus. Sci Rep. (2020) 10:5851. doi: 10.1038/s41598-020-62480-3 32245990 PMC7125190

[22] Lande R Pietraforte I Mennella A Palazzo R Spinelli FR Giannakakis K. Complementary effects of carbamylated and citrullinated LL37 in autoimmunity and inflammation in systemic lupus erythematosus. Int J Mol Sci. (2021) 22:1650. doi: 10.3390/ijms22041650 33562078 PMC7915858

[23] Scherer HU van der Woude D Toes REM. From risk to chronicity: evolution of autoreactive B cell and antibody responses in rheumatoid arthritis. Nat Rev Rheumatol. (2022) 18:371–83. doi: 10.1038/s41584-022-00786-4 35606567

[24] Chiang CC Cheng WJ Korinek M Lin CY Hwang TL. Neutrophils in psoriasis. Front Immunol. (2019) 10:2376. doi: 10.3389/fimmu.2019.02376 31649677 PMC6794444

[25] Wang WM Jin HZ. Role of neutrophils in psoriasis. J Immunol Res. (2020) 2020:3709749. doi: 10.1155/2020/3709749 32587871 PMC7293746

[26] Pietraforte I Butera A Gaddini L Mennella A Palazzo R Campanile D. CXCL4-RNA complexes circulate in systemic sclerosis and amplify inflammatory/pro-fibrotic responses by myeloid dendritic cells. Int J Mol Sci. (2023) 24:653. doi: 10.3390/ijms.24010653 PMC9820649 36614095

[27] Frasca L Nasso M Spensieri F Fedele G Palazzo R Malavasi F. IFN-γ arms human dendritic cells to perform multiple effector functions. J Immunol. (2008) 180:1471–81. doi: 10.4049/jimmunol.180.3.1471 18209042

[28] Harder J Schroder JM. Psoriatic scales: a promising source for the isolation of human skin-derived antimicrobial proteins. J Leukoc Biol. (2005) 77:476–86. doi: 10.1189/jlb.0704409 15629886

[29] Lande R Palazzo R Hammel P Pietraforte I Surbeck I Gilliet M. Generation of monoclonal antibodies specific for native LL37 and citrullinated LL37 that discriminate the two LL37 forms in the skin and circulation of cutaneous/systemic lupus erythematosus and rheumatoid arthritis patients. Antibodies. (2020) 9:14. doi: 10.3390/antib9020014 32403306 PMC7345132

[30] Eneida V Srilakshmi Y Celine CB Jeffrey BH Ritika K Andrew ML. Netting neutrophils induce endothelial damage, infiltrate tissues, and expose immunostimulatory molecules in systemic lupus erythematosus. J Immunol 1 July. (2011) 187:538–52. doi: 10.4049/jimmunol.1100450 PMC3119769 21613614

[31] Brinkmann V Abu Abed U Goosmann C Zychlinsky A. Immunodetection of NETs in paraffin-embedded tissue. Front Immunol. (2016) 7:513. doi: 10.3389/fimmu.2016.00513 27920776 PMC5118445

[32] Schoenfeld L Appl B Pagerols-Raluy L Heuer A Reinshagen K Boettcher M. Immunofluorescence imaging of neutrophil extracellular traps in human and mouse tissues. J Vis Exp. (2023) 198):e65272. doi: 10.3791/65272 37677039

[33] Zeneng W Stephen JN Rodriguez ER Outi K Sohvi Hörkkö JB Wanda F. Protein carbamylation links inflammation, smoking, uremia and atherogenesis. Natu Med. (2007) 10):1176–84. doi: 10.1038/nm1637 17828273

[34] Shuichiro N Koichiro O Shuji A Kosaku M Ran N Motomu H. Activated neutrophil carbamylates albumin via the release of myeloperoxidase and reactive oxygen species regardless of NETosis. Modern Rheumatol. (2020) 2):345–9. doi: 10.1080/14397595.2019.1583819 30789095

[35] Martín Monreal MT Kvist-Hansen A Massarenti L Steffensen R Loft N Hansen PR. Characterization of circulating extracellular traps and immune responses to citrullinated LL37 in psoriasis. Front Immunol. (2023) 14:1247592. doi: 10.3389/fimmu.2023.1247592 38173716 PMC10762777

[36] Wang Y Wang L Yang H Yuan W Ren J Bai Y. Activated circulating T follicular helper cells are associated with disease severity in patients with psoriasis. J Immunol Res. (2016) 2016:7346030. doi: 10.1155/2016/7346030 27774460 PMC5059604

[37] Vinuesa CG Linterman MA Yu D MacLennan IC. Follicular helper T cells. Annu Rev Immunol. (2016) 34:335–68. doi: 10.1146/annurev-immunol-041015-055605 26907215

[38] Caruso R Botti E Sarra M Esposito M Stolfi C Diluvio L. Involvement of interleukin-21 in the epidermal hyperplasia of psoriasis. Nat Med. (2009) 15:1013–15. doi: 10.1038/nm.1995 19684581

[39] Brinkmann V Geiger T Alkan S Heusser CH. Interferon alpha increases the frequency of interferon gamma-producing human CD4+ T cells. J Exp Med. (1993) 178:1655–63. doi: 10.1084/jem.178.5.1655 PMC2191249 8228812

[40] Nakayamada S Poholek AC Lu KT Takahashi H Kato M Iwata S. Type I IFN induces binding of STAT1 to Bcl6: divergent roles of STAT family transcription factors in the T follicular helper cell genetic program. J Immunol. (2014) 192:2156–66. doi: 10.4049/jimmunol.1300675 PMC3967131 24489092

[41] Strengell M Julkunen I Matikainen S. IFN-α regulates IL-21 and IL-21R expression in human NK and T cells. J Leukoc Biol. (2004) 76:416–22. doi: 10.1189/jlb.1003488 15178704

[42] Ray JP Marshall HD Laidlaw BJ Staron MM Kaech SM Craft J. Transcription factor STAT3 and type I interferons are corepressive insulators for differentiation of follicular helper and T helper 1 cells. Immunity. (2014) 40:367–77. doi: 10.1016/j.immuni.2014.02.005 PMC3992517 24631156

[43] Leon B Lopez-Bravo M Ardavin C. Monocyte-derived dendritic cells. Semin Immunol. (2005) 17:313–8. doi: 10.1016/j.smim.2005.05.013 15955712

[44] Nestle FO Conrad C Tun-Kyi A Homey B Gombert M Boyman O. Plasmacytoid predendritic cells initiate psoriasis through interferon-alpha production. J Exp Med. (2005) 202:135–43. doi: 10.1084/jem.20050500 PMC2212894 15998792

[45] Zhou Y Chen B Mittereder N Chaerkady R Strain M An LL. Spontaneous secretion of the citrullination enzyme PAD2 and cell surface exposure of PAD4 by neutrophils. Front Immunol. (2017) 25:1200. doi: 10.3389/fimmu.2017.01200 PMC5622307 28993780

[46] Boeltz S Amini P Anders HJ Andrade F Bilyy R Chatfield S. To NET or not to NET:current opinions and state of the science regarding the formation of neutrophil extracellular traps. Cell Death Differ. (2019) 3):395–408. doi: 10.1038/s41418-018-0261-x PMC6370810 30622307

[47] Romero V Fert-Bober J Nigrovic PA Darrah E Haque UJ Lee MD. Immune-mediated pore-forming pathways induce cellular hypercitrullination and generate citrullinated autoantigens in rheumatoid arthritis. Sci Transl Med. (2013) 5:209ra150. doi: 10.1126/scitranslmed.3006869 PMC4032227 24174326

[48] Lambert S Hambro CA Johnston A Stuart PE Tsoi LC Nair RP. Neutrophil extracellular traps induce human th17 cells: effect of psoriasis-associated TRAF3IP2 genotype. J Invest Dermatol. (2019) 139:1245–53. doi: 10.1016/j.jid.2018.11.021 PMC7092801 30528823

[49] Di Domizio J Gilliet M. Psoriasis caught in the NET. J Invest Dermatol. (2019) 139:1426–9. doi: 10.1016/j.jid.2019.04.020 31230639

[50] Herster F Bittner Z Archer NK Dickhöfer S Eisel D Eigenbrod T. Neutrophil extracellular trap-associated RNA and LL37 enable self-amplifying inflammation in psoriasis. Nat Commun. (2020) 11:105. doi: 10.1038/s41467-019-13756-4 31913271 PMC6949246

[51] Bork F Greve CL Youn C Chen S Leal V NC Wang Y. naRNA-LL37 composite DAMPs define sterile NETs as self-propagating drivers of inflammation. EMBO Rep. (2024) 7):2914–49. doi: 10.1038/s44319-024-00150-5 PMC11239898 38783164

[52] Li R Xiong Y Ma L Peng C Qi S Gao R. Neutrophil extracellular traps promote macrophage inflammation in psoriasis. Clin Immunol. (2024) 266:110308. doi: 10.1016/j.clim.2024.110308 39002794

[53] Shao S Fang H Dang E Xue K Zhang J Li B. Neutrophil extracellular traps promote inflammatory responses in psoriasis via activating epidermal TLR4/IL-36R crosstalk. Front Immunol. (2019) 10:746. doi: 10.3389/fimmu.2019.00746 31024570 PMC6460719

[54] Ganguly D Chamilos G Lande R Gregorio J Meller S Facchinetti V. Self-RNA-antimicrobial peptide complexes activate human dendritic cells through TLR7 and TLR8. J Exp Med. (2009) 206:1983–94. doi: 10.1084/jem.20090480 PMC2737167 19703986

[55] Wong A Bryzek D Dobosz E Scavenius C Svoboda P Rapala-Kozik M. A novel biological role for peptidyl-arginine deiminases: citrullination of cathelicidin LL-37 controls the immunostimulatory potential of cell-free DNA. J Immunol. (2018) 200:2327–40. doi: 10.4049/jimmunol.1701391 PMC5860981 29475987

[56] Lande R Ganguly D Facchinetti V Frasca L Conrad C Gregorio J. Neutrophils activate plasmacytoid dendritic cells by releasing self-DNA-peptide complexes in systemic lupus erythematosus. Sci Transl Med. (2011) 3:73ra19. doi: 10.1126/scitranslmed.3001180 PMC3399524 21389263

[57] Sun S Duan Z Wang X Chu C Yang C Chen F. Neutrophil extracellular traps impair intestinal barrier functions in sepsis by regulating TLR9-mediated endoplasmic reticulum stress pathway. Cell Death Dis. (2021) 12:606. doi: 10.1038/s41419-021-03896-1 34117211 PMC8195983

[58] Li M Liu Y Wang J Wang Y Yang Y Yang A. Neutrophil extracellular DNA traps activate the TLR9 signaling pathway of pancreatic ductal epithelial cells in patients with type 2 autoimmune pancreatitis. Int Immunopharmacol. (2025) 144:113673. doi: 10.1016/j.intimp.2024.113673 39616853

[59] Garcia-Romo GS Caielli S Vega B Connolly J Allantaz F Xu Z. Netting neutrophils are major inducers of type I IFN production in pediatric systemic lupus erythematosus. Sci Transl Med. (2011) 3:73ra20. doi: 10.1126/scitranslmed.3001201 PMC3143837 21389264

[60] Lood C Blanco LP Purmalek MM Carmona-Rivera C De Ravin SS Smith CK. Neutrophil extracellular traps enriched in oxidized mitochondrial DNA are interferogenic and contribute to lupus-like disease. Nat Med. (2016) 2):146–53. doi: 10.1038/nm.4027 PMC4742415 26779811

[61] Konig MF Andrade F. A critical reappraisal of neutrophil extracellular traps and NETosis mimics based on differential requirements for protein citrullination. Front Immunol. (2016) 7:461. doi: 10.3389/fimmu.2016.00461 27867381 PMC5095114

[62] Wang S Wu M Chiriboga L Zeck B Belmont HM. Membrane attack complex (mac) deposition in lupus nephritis is associated with hypertension and poor clinical response to treatment. Semin Arthritis Rheumatol. (2018) 2):256–62. doi: 10.1016/j.semarthrit.2018.01.004 29395256

[63] Pietraforte I Frasca L. Autoreactive T-Cells in Psoriasis: are they spoiled Tregs and can therapies restore their functions? Int. J Mol Sci. (2023) 24:4348. doi: 10.3390/ijms24054348 PMC10002349 36901778

[64] Chen Y Yu D. TCF-1 at the tfh and th1 divergence. Trends Immunol. (2015) 36:758–60. doi: 10.1016/j.it.2015.11.001 26588880

[65] Zhang J Feng Y Shi D. NETosis of psoriasis: a critical step in amplifying the inflammatory response. Front Immunol. (2024) 15:1374934. doi: 10.3389/fimmu.2024.1374934 39148738 PMC11324545

[66] Zhang ZH Zhan ZY Jiang M Wang XY Quan SL Wu YL. Casting NETs on Psoriasis: The modulation of inflammatory feedback targeting IL-36/IL-36R axis. Int Immunopharmacol. (2024) 142:113190. doi: 10.1016/j.intimp.2024.113190 39306890

[67] Janke M Poth J Wimmenauer V Giese T Coch C Barchet W. Selective and direct activation of human neutrophils but not eosinophils by Toll-like receptor 8. J Allergy Clin Immunol. (2009) 123:1026–33. doi: 10.1016/j.jaci.2009.02.015 19361845

[68] Reich K Papp KA Matheson RT Tu JH Bissonnette R Bourcier M. Evidence that a neutrophil-keratinocyte crosstalk is an early target of IL-17A inhibition in psoriasis. Exp Dermatol. (2015) 7):529–35. doi: 10.1111/exd.12710 PMC4676308 25828362

[69] Takagi N Kawakami K Kanno E Tanno H Takeda A Ishii K. IL-17A promotes neutrophilic inflammation and disturbs acute wound healing in skin. Exp Dermatol. (2017) 26:137–44. doi: 10.1111/exd.131152 27305096

[70] Mitchell TS Moots RJ Wright HL. Janus kinase inhibitors prevent migration of rheumatoid arthritis neutrophils towards interleukin-8, but do not inhibit priming of the respiratory burst or reactive oxygen species production. Clin Exp Immunol. (2017) 2):250–8. doi: 10.1111/cei.12970 PMC5508336 28369741

[71] Paik J Deeks ED. Tofacitinib: A review in psoriatic arthritis. Drugs. (2019) 6):655–63. doi: 10.1007/s40265-019-01091-3 30895473

